# Establishing a reference cultivation protocol for systematic characterisation of the *Halomonas* genus

**DOI:** 10.3389/fbioe.2026.1923905

**Published:** 2026-09-24

**Authors:** Waritthorn Thanakarn, Alexander Antoine Maria Baumbach, Eva Price, Duygu Dikicioglu

**Affiliations:** Department of Biochemical Engineering, University College London, London, United Kingdom

**Keywords:** culture temperature, growth physiology, *Halomonas*, high-throughput screening, LB medium, salt concentration, specific growth rate

## Abstract

**Introduction:**

Halomonas species are increasingly recognised as promising next‐generation industrial biotechnology chassis due to their capacity to grow under high-salt, non-sterile conditions, reducing contamination risk and eliminating the need for resource‐intensive sterilisation. Despite this potential, most Halomonas species remain poorly characterised beyond initial isolation, limiting their adoption. Developing reference cultivation conditions for Halomonas genus, which could be employed as standard practice, is a prerequisite for extended biotechnological and industrial uptake.

**Methods:**

The maximum OD600 and maximum specific growth rates of 82 Halomonas strains representing 80 species were determined under their respective culture collection‐recommended conditions, encompassing 27 unique sets of cultivation parameters.

**Results and Discussion:**

A subsequently developed reference cultivation protocol supported growth of 95% of strains in Luria‐Bertani (LB) broth at either low‐ (2% NaCl; LB20) or moderate-salt (8% NaCl; LB80) concentrations at either 28 °C or 37 °C. Of the remaining 78 strains, only two performed significantly worse in both specific growth yield and maximum OD600 when a reference protocol was followed. Twenty‐three strains were tested for growth at either 28 °C or 37 °C due to the differing recommended temperatures by the strain providers. Transition to salt-supplemented LB broth significantly increased specific growth rates in ten strains and maximum OD600 in eleven strains. Of the 82 strains, 67 were capable of growth in moderate‐salt LB broth compared to only 29 in low‐salt LB broth, demonstrating that elevated salinity is broadly permissive across the genus. With the exception of four strains, all members of the in‐house collection were able to grow when a reference protocol was followed. The proposed reference culture conditions were LB80 at 28 °C for 35 strains, LB80 at 37 °C for 14 strains, either LB80 or LB20 at 28 °C for 13 strains, LB20 at 28 °C for ten strains, either LB80 or LB20 at 37 °C for five strains, and LB20 at 37 °C for one strain. This cultivation framework, comprising LB medium supplemented with 2% or 8% NaCl at 28 °C or 37 °C, is broadly applicable across the Halomonas genus and provides a standardised, resource‐efficient foundation for systematic strain characterisation, comparative physiological studies, and future engineering efforts aimed at expanding the industrial utility of this promising microbial chassis.

## 1 Introduction

*Halomonas* species emerge as a non-model bacterial chassis with a number of potential industrial uses (Yan et al., 2025). Their high salt tolerance renders them advantageous for long-term fermentation, minimising contamination risk (Yoo et al., 2024) and reducing the need for sterilisation (Zhang et al., 2024). Consistent with this rationale, open and non-sterile fermentation of *Halomonas campaniensis* LS21 for the production of polyhydroxyalkanoates (PHAs) was reported to operate continuously and successfully for 65 days (Yue et al., 2014). The use and optimisation of seawater as a substrate was explored, too, for *Halomonas* spp. Further demonstrating its potential for sustainable bioprocessing (Zhou et al., 2021).

*Halomonas* species are gram-negative, rod-shaped bacteria capable of thriving in both aerobic and anaerobic environments (Bogale and Aragaw, 2025). *Halomonas elongata* was the first species isolated and identified as a *Halomonas* species (Vreeland et al., 1980; Tsuji et al., 2022); as of 2023, nearly 120 species were confirmed to be associated with the genus (Kim and Park, 2023). Currently, 130 *Halomonas* species were reported in the List of Prokaryotic names with Standing in Nomenclature (Freese et al., 2026).

Halophilic bacteria can grow at different levels of salt concentration ranging from low (2%–5%) to moderate (5%–20%) to high (20%–30%) (Yoo et al., 2023). They possess two primary adaptation mechanisms to withstand high salinity environments: the salt-in approach, where the bacteria accumulate inorganic salts or ions, such as potassium (K^+^), within their cytosol (Saini et al., 2023), and the synthesis or absorption of suitable solutes, called the osmolytes, to establish osmotic equilibrium within the cell and preserve protein structure and stability (Gregory and Boyd, 2021; Abosamaha et al., 2022; Yu et al., 2024). Among the two economically relevant products that *Halomonas* can synthesise, ectoine, 1,4,5,6-tetrahydro-2-methyl-4-pyrimidinecarboxylic acid, serves as their principal compatible osmolyte (Gunde-Cimerman et al., 2018). Ectoine is effectively utilised in pharmaceutical and cosmetic applications due to its potential to safeguard proteins and other macromolecules under extreme conditions such as high salinity, elevated temperatures, or ionising radiation (Hobmeier et al., 2022).

Another key product group synthesised by *Halomonas* spp. are PHAs typically adopted as energy and carbon storage mechanisms in nutrient-limited environments (Xie et al., 2024). PHAs are classified into bioplastics, and are considered a sustainable alternative to petroleum-based polymers (Hendy et al., 2025). The economic relevance of both products led to intensive engineering biology efforts for yield and process improvement, examples of which include the utilisation of CRISPR/Cas9 technology to disrupt *hom* gene to increase ectoine production in *H. campaniensis* XH26 (Shu et al., 2023) or the modification of *Halomonas cupida* J9 to synthesise PHA from maize straw hydrolysate (Wang et al., 2024).

Furthermore, *Halomonas* species were metabolically engineered to produce industrially relevant non-native products such as mevalonate (Zhang et al., 2023) or lycopene (Su et al., 2022). Beyond product biosynthesis, their fitness under hypoxic conditions was improved through adaptive laboratory evolution (Thanakarn et al., 2026). These findings collectively support the potential of *Halomonas* as a viable alternative microbial chassis for industrial biotechnology.

Luria-Bertani, also referred to as Lysogeny broth (LB) medium, is a rich and simple medium that allows many bacterial species to grow rapidly, reaching competent biomass yields (Sezonov et al., 2007). It is the medium of choice for early-stage bacterial culturing, particularly owing to their capacity to support monoculture growth and their adoption in molecular biology protocols (Tan et al., 2014; Ma et al., 2020). Consequently, it was also adopted to support the growth of recently isolated *Halomonas* strains (Tsuji et al., 2022; Christensen et al., 2025) as well as in the preparation of seed cultures for preliminary bioprocess studies (Lan et al., 2016; Zhao et al., 2019). Despite this practical utility, a plethora of different growth conditions were recommended for different species and strains of the *Halomonas* genus in globally acknowledged culture collections such as DSMZ and ATCC. Unlike model organisms such as *Escherichia coli* and *Saccharomyces cerevisiae*, which also serve as the type species of their respective genera, multitude of species of the *Halomonas* genus are simultaneously explored and utilised for specific biotechnological applications. This necessitates the establishment of a reference set of growth conditions that would be recommendable to support the fitness of multiple species to allow comparability as well as to streamline *Halomonas*-specific protocol development and lab practice, in general.

We address this critical current gap in the field and propose a reference cultivation protocol capable of supporting the growth of 82 *Halomonas* strains representing 80 species sourced from accessible culture collections (representing ca. 62% of the genus) and is the broadest genus-wide cultivation and fitness assessment to date. The protocol is intentionally designed to be simple and resource-efficient, employing cost-effective media supplemented with modest salt concentrations that preserve the hallmark salt-tolerant characteristics of the genus while minimising corrosive burden on equipment and infrastructure. Crucially, conditions were established through a systematic, stepwise transition from the collection-recommended medium for each strain, modifying one parameter at a time (medium composition, temperature, and salt concentration, see Figure 1) and evaluating its impact on growth fitness at each stage. While this protocol is not intended to maximise the performance of individual strains under tightly controlled bioprocess conditions, it provides a standardised entry point for preliminary strain characterisation and synthetic biology workflows.

**FIGURE 1:**
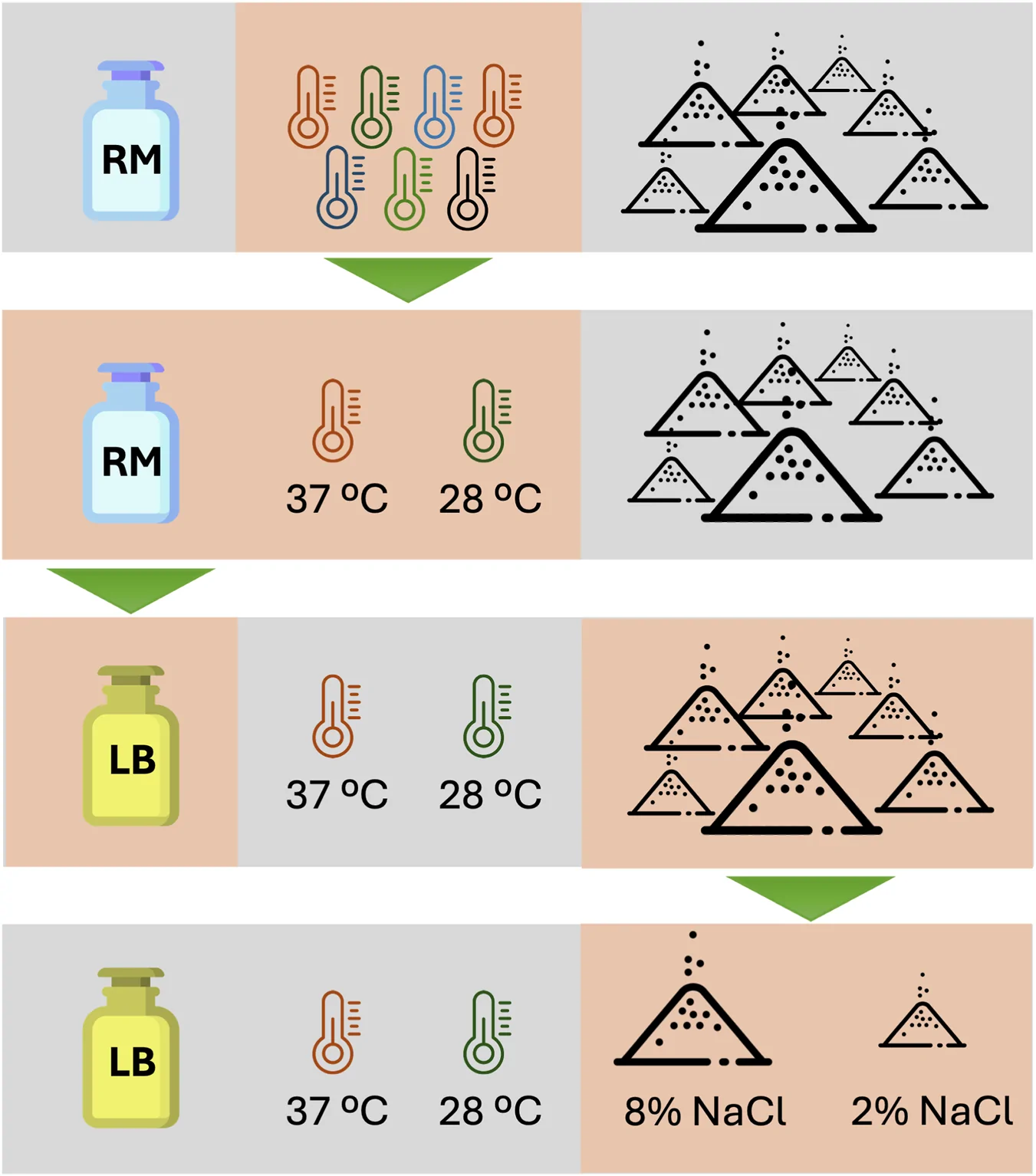
Stepwise consolidation of culture collection-recommended cultivation conditions into a reference protocol for *Halomonas* species. The 27 distinct cultivation conditions recommended across the 82 strains investigated were systematically transitioned into one of four reference conditions through sequential evaluation of three parameters: temperature, medium composition, and NaCl concentration, assessing the impact of each transition on specific growth rate and maximum OD_600_. The resulting reference conditions suggest cultivation at 28 °C or 37 °C in LB broth supplemented with either 2% or 8% NaCl. Icons sourced from Flaticon.com.

## 2 Materials and methods

### 2.1 *Halomonas* strain collection and culture media

A total of 82 strains of *Halomonas* species were procured in 2023 from the Leibniz Institute, DSMZ-German Collection of Microorganisms and Cell Cultures GmbH (DSMZ, Germany) and American Type Culture Collection (ATCC) (Supplementary Table S1). Freeze-dried cells were resuspended in their respective recommended media, as reported on the DSMZ and ATCC websites. Following the initial revival of the strains and preparation of working stocks, all experimental cultivations were performed using these working stocks under consistent cultivation and handling procedures throughout the study. No strains were re-revived from the original ampoules during the experimental period. In this study, all strains’ catalogue numbers from DSMZ and ATCC were referred to for traceability due to some strains lacking designation. The recipes for Medium 1034, Medium 1689, Medium 2049, Medium 830, Medium 372, and Medium TSD-110 are provided in Supplementary Table S2. Media were supplemented with sodium chloride (Reagent Plus® ≥99%, Sigma-Aldrich, Cat. No. S9625) as recommended (Supplementary Table S1). Marine Broth 2216 (Merck Millipore, Cat. No. 76448), Difco™ Marine Broth 2216 (BD, Cat. No. 279110), and LB-Broth, high salt medium (Merck Millipore, Cat. No. 51208) were purchased as ready-to-use powder. For the preparation of the reference medium, LB-Broth, high salt medium (10 g⋅L^-1^ casein enzymic hydrolysate, 10 g⋅L^-1^ sodium chloride, 5 g⋅L^-1^ yeast extract) was supplemented with NaCl to achieve a final salt concentration of 20 g⋅L^-1^ (2%) and 80 g⋅L^-1^ (8%); these media are denoted as LB20 and LB80, respectively. A salinity of 8% NaCl was selected for the reference cultivation medium based on both the culture collections’ recommended growth conditions and prior experimental evidence. Among the 82 strains investigated in this study, 39 were recommended for growth at salinities above 5%; of these, 18 were recommended to be grown at 10% salinity and 19 at 8% salinity. In addition, our previous work (Thanakarn et al., 2026) found no measurable difference in contamination resistance between 9.5% and 8% NaCl. Taken together, these observations support 8% NaCl as a suitable and representative salinity for the reference cultivation protocol.

Mainly, Medium 514 in this study referred to Marine Broth 2216 and was used for all 82 strains, apart from *Halomonas muralis* DSM14789, *Halomonas kribbensis* DSM17892, *Halomonas urmiana* DSM22871, *Halomonas tabrizica* DSM23018, *Halomonas koreensis* DSM23530, *Halomonas saccharevitans* DSM23548, and *Halomonas maris* DSM112092, which were cultured in Difco^TM^ Marine Broth 2216.

### 2.2 *Halomonas* growth cultivation

All *Halomonas* species were pre-cultured from −80 °C glycerol stock in their recommended media (RM), LB with the corresponding recommended salt concentration (LBS), LB20 or LB80 medium overnight, except for 22% of the strain collection showing no growth within this window, an extended cultivation period of 14 days was applied. *Halomonas subglaciescola*, *Halomonas maura*, *H*. *muralis*, *Halomonas campisalis*, *Halomonas gomseomensis*, *Halomonas korlensis*, *Halomonas ilicicola*, *Halomonas sediminis*, *Halomonas vilamensis*, *H*. *urmiana*, *H*. *tabrizica*, *Halomonas nitroreducens*, *H*. *koreensis*, *H*. *saccharevitans*, *Halomonas arcis*, *Halomonas almeriensis*, *H*. *maris*, and *H*. *kribbensis* were able to grow in at least one of these conditions within the stated timeframe of 2 weeks confirming the required additional time when taken out of the glycerol stocks to confer a healthy preculture. It should be noted that once the cultures were inoculated from these precultures, the culturing timeframes for these cells no longer necessitated to get outside of the range described below. For the high-throughout experiments, cells were inoculated in fresh medium at an initial OD_600_ of 0.05. Fresh cell cultures were then aliquoted at 200 μL per well (1:2 fill volume to well volume) in a 96-microwell plate (SARSTEDT, Cat. No. 82.1581) in six biological replicates (n = 6). Plates were covered with Aeraseal^TM^ (EXCEL, Cat. No. Z721573) and incubated in CLARIOstar Plus plate reader (BMG Labtech) operated at a rotational agitation of 200 rpm (double orbital shaking), set at different temperatures (22 °C–40 °C) for at least 46 h, up to 82 or 136 h until stationary phase. During the cultivation, pH and aeration were not controlled in the plate reader. Because oxygen transfer may vary across different salt compositions in the culture media, Aeraseal sealing membranes were used to maximise aeration capacity. Prior to the design of the full-scale cultivation study, a dedicated technical validation was performed to rule out positional bias across the microwell plate, given the extended incubation times (46–136 h) and the potential for evaporation or edge effects under agitated, gas-permeable-sealed conditions. Forty-five replicate wells were run in parallel, with the remaining wells serving as controls, and this experiment was repeated on a second plate to assess inter-plate consistency. Wells were classified into three position categories: peripheral, adjacent-to-peripheral, and innermost-centre, and pairwise comparisons of specific growth rate and maximum OD_600_ were performed across all three category pairs. No significant differences were observed for any comparison, on either plate (all p > 0.05), confirming the absence of measurable edge- or evaporation-driven positional effects under the incubation conditions used in this study. The optical density (OD_600_) was measured every 5 min for cultures lasting 82 h and every 10 min for cultures lasting 136 h, and used for growth rate calculation and for the identification of maximum OD_600_ (OD_max_).

### 2.3 Specific growth rate calculation and maximum OD_600_ determination

The first OD_600_ reading was normalised to OD_600_ = 0.05, treating it as an additive offset; the same adjustment was applied to all following points. Medium blanks were handled per condition, each growth curve was corrected using the blank corresponding to its own medium (LB or the relevant collection medium at relevant salt concentration), measured in parallel wells during the same run and subtracted automatically by the CLARIOstar software. This condition-specific blank correction also accounts for baseline optical differences between media of differing salt composition, since any refractive-index-related offset present in the uninoculated medium is captured and subtracted at the level of each individual well prior to growth rate and OD_max_ determination. OD_600_ readings were natural logarithm-transformed (ln (OD_600_)) and were plotted against cultivation time (h). The linear range denoting the exponential phase of each strain was selected from the plots ensuring that the timeframe covers at least two hours. The slope from the linear range of the plot of ln (OD_600_) against the incubation time was determined as the specific growth rate (μ). Coefficient of determination (R^2^) was ensured to be at least 0.8 or above. The R^2^ ≥ 0.8 threshold was chosen to be permissive enough to accommodate the range of curve shapes observed during the exponential phase specifically, rather than assuming a uniform degree of fit utilising the conventional R^2^ ≥ 0.95 benchmark across all strains. Consistent with the variability in growth curves, the coefficient of determination was reported to be above the benchmark of 0.95 in only 34.2% of the μ calculations. We further note that plate-reader absorbance measurements are subject to non-linearity at high optical densities, a general and well-recognised limitation of turbidimetric growth assays performed on plate readers, including the CLARIOstar Plus used in this study, rather than one specific to the present experimental design. Readings above the linear range were used as recorded, without dilution or correction, consistent with common practice in comparable growth assay studies employing CLARIOstar and similar plate-reader instruments. Growth rates of all replicate runs were calculated. The highest OD_600_ reading was reported as OD_max_ in each run. The average and the standard deviation of μ and OD_max_ of each strain were calculated and reported per strain. Other orthogonal validations, such as dry cell weight, colony forming unit (CFU) counts, and metabolic viability, were not performed in this study. Generating such calibration data across both LB20 and LB80 conditions for even a subset of strains would require substantial additional biomass per condition, which is incompatible with the 200 μL microwell format used to screen 82 strains × 6 biological replicates across recommended, reference, and intermediate cultivation conditions. High-throughput OD-based growth screening using microplate readers is well established in the microbiology literature as a validated approach for generating high-resolution kinetic data at a scale unattainable by direct biomass methods, with the accepted understanding that OD_600_ serves as a proxy measure rather than a direct biomass readout.

### 2.4 Phylogenetic tree and hierarchical clustering analysis

A phylogenetic tree of 79 *Halomonas* strains was constructed from 16S rRNA sequences (accession numbers in Supplementary Table S3). Sequences were aligned using MAFFT (v7.0.0) and trimmed using trimAl (v1.5.0). Maximum-likelihood inference was performed in IQ-TREE (v3.0.1) with model selection via ModelFinder and branch support assessed using SH-aLRT and Ultrafast Bootstrap (1,000 replicates each). The tree was rooted using the midpoint method against explicit outgroup rooting (*Zymobacter palmae*), which recovered an identical topology. Tree-growth phenotype comparisons (hierarchical clustering, tanglegram analysis, Mantel tests, Blomberg’s K and Pagel’s λ) were performed in R studio (v2025.9.1.401) with R (v4.5.1). Full methodological detail are provided in Supplementary Analysis S1 and the version information is summarised in Supplementary Table S4.

### 2.5 Bioreactor settings in batch cultivation

DASbox® Mini Bioreactor System (Eppendorf) was used for batch bioreactor runs, all conditions run in triplicates. *Halomonas stevensii*, *H*. *elongata* and *Halomonas magadiensis* were retrieved from the glycerol stock and were pre-cultured in 10 mL LB medium in 50 mL Falcon^TM^ centrifuge tubes using the LB-based reference protocol for 24–48 h. Seed cultures (50 mL reference medium in 250 mL shake flask) were incubated at 28 °C (*H*. *stevensii* and *H*. *elongata)* or 37 °C (*H*. *magadiensis*) with orbital shaking at 200 rpm overnight. The bioreactor vessels were inoculated to 0.05 initial OD_600_ in 170 mL working volume of LB20 or LB80. The temperature was set at 28 °C for *H*. *stevensii* and *H*. *elongata*, and at 37 °C for *H*. *magadiensis*. The pH was controlled at 7.0 using 8%–12% phosphoric acid and ammonium solution. The dissolved oxygen was set at 30% and controlled by supplementing air and modifying the agitation speed between 400 rpm (initial speed) to 1,000 rpm (maximum allowable speed) based on a pre-defined regime. Polypropylene glycol was used as antifoam and was added 6 h post-inoculation. Bioreactors were sampled at 6, 24, and 49 h post-inoculation for OD_600_ measurement.

### 2.6 Statistical analysis

All data presented in this research was reported as average ±standard deviation (SD). A two-tailed unpaired t-test analysis was conducted for statistical significance analysis, assuming equal variance between groups, by Microsoft Excel to determine the 95% (p < 0.05) and 99% (p < 0.01) confidence intervals. The equal variance assumption was then challenged by conducting Welch’s t-test, which yielded very similar p-values to prior analysis confirming near equal variance assumption between groups. The analysis was performed by comparing two different conditions per strain, using six biological replicates (n = 6). A Benjamini Hochberg FDR threshold of q = 0.10 was employed and the reported p-value threshold throughout the results represents an adjusted p-value. Graphs and diagrams were generated using Microsoft Excel and GraphPad Prism software 10 for Mac (GraphPad Software, Boston, Massachusetts United States (www.graphpad.com). Venn diagram was generated by Venny 2.1 (Oliveros, 2007).

## 3 Results

### 3.1 Growth characterisation of 82 *Halomonas* strains under culture collection-recommended conditions

A total of 82 *Halomonas* strains available through DSMZ or ATCC were employed in this study. Seventy-six strains were classified as type strains, while three strains, namely, *Halomonas glaciei* DSM15938, *Halomonas lionensis* DSM25632, and *Halomonas zhaodongensis* DSM25869 were designated as proposed type strains (Supplementary Table S1) for their respective species. On the other hand, *Halomonas* sp. DSM7328, *Halomonas alkaliantarctica* DSM15686, and *Halomonas caseinilytica* DSM18067 were not reported as type strains of their respective species. Two of these species were investigated by two strains each, one of which was a type strain. *H. caseinilytica* DSM18067 and *H. caseinilytica* DSM23509 were isolated from distinct sources and were reported to grow in different recommended temperatures and salt concentrations. Likewise, *Halomonas lutea* DSM19221 and *H. lutea* DSM23508 were recommended to grow at different salt concentrations and were therefore considered to be of particular interest for the purposes of this study (Supplementary Table S1). In summary, this collection was comprised of 80 species, offering the opportunity to investigate the genomic and cognate phenotypic characterisation of the members of the genus at scale. For this purpose, we constructed the most comprehensive phylogenetic tree of 78 *Halomonas* species to date in order to identify and elucidate the genotypic similarities and differences among the species in this collection (Figure 2A). Two species, *Halomonas* sp. DSM7328 and *Halomonas eurialkalitoleransis* TSD-110, were excluded from this phylogenetic tree due to current lack of availability of 16S rRNA sequences retrievable from public repositories. Additionally, *H. lutea* DSM19221 lacks an independent 16S rRNA sequence and is represented on the tree by the sequence of *H. lutea* DSM23508 (Supplementary Table S3).

**FIGURE 2:**
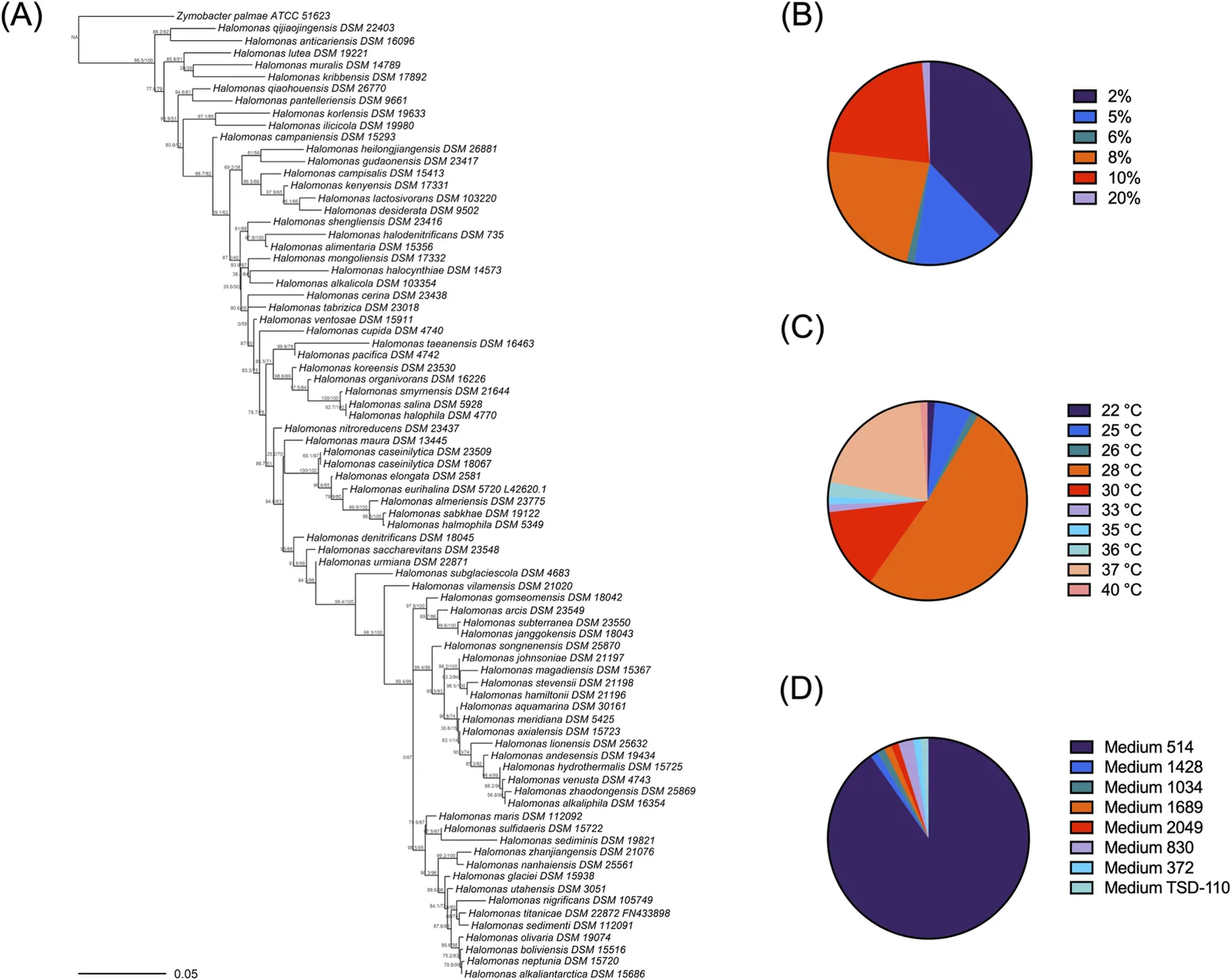
Phylogenetic and cultivation condition diversity across the Halomonas strains investigated in this study. **(A)** Phylogenetic tree constructed from 16S rRNA sequences retrieved from the NCBI database. Culture collection-recommended conditions are summarised with respect to **(B)** NaCl concentration, across six levels ranging from 2% to 20%, **(C)** recommended cultivation temperature, spanning ten values between 22 °C and 40 °C, and **(D)** growth medium composition with eight unique culture media.

Based on the recommendations of the DSMZ and ATCC culture collections, the majority of the strains were typically cultured in the presence of either 2% (31 strains) or 8% (19 strains) salt (Figure 2B) and grown at 28 °C (42 strains) or 37 °C (17 strains) (Figure 2C). The culture media recommended for different *Halomonas* strains was also guided by the diverse ecological niches and environments that these strains were isolated from; Marine broth 2216 and DSMZ Medium514 were the most frequently reported media for supporting *Halomonas* growth (Figure 2D).

The average specific growth rate of all strains cultured in their respective recommended conditions was 0.222 ± 0.116 h^-1^. *Halomonas halmophila* DSM5349 had the lowest specific growth rate (µ = 0.030 ± 0.006 h^-1^) and *Halomonas hydrothermalis* DSM15725 was determined to have the highest specific growth rate (µ = 0.537 ± 0.151 h^-1^). The average of the maximum OD_600_ attainable by these strains was 2.610 ± 1.048 when cultured under their respective recommended conditions. The lowest maximum OD_600_ value was recorded for *H. gomseomensis* DSM18042 (OD_600_ = 0.702 ± 0.274), and the highest maximum OD_600_ value was recorded for *Halomonas meridiana* DSM5425 (OD_600_ = 3.869 ± 0.202). The collection-recommended medium was not able to support the growth of *Halomonas janggokensis* DSM18043 with an apparent growth rate very close to zero consistently, achieving minimal growth approaching a maximum OD_600_ of 0.320 ± 0.130 after 81 h (Supplementary Table S5). *H. janggokensis* growth was subsequently recovered when cultured in LB medium.

### 3.2 Towards two reference growth temperatures for *Halomonas* strains

The 82 *Halomonas* strains examined in this study were isolated from geographically and ecologically diverse environments, reflected in their collection-recommended growth temperatures spanning a range from 22 °C to 40 °C. As reported above, the collection-recommended growth temperatures for the majority of strains converged to either 28 °C or 37 °C. The remaining 23 strains, recommended at intermediate or divergent temperatures, were therefore assessed for their ability to grow at either condition, with the aim of identifying strains exhibiting minimal adverse temperature sensitivity that could be accommodated within this binary framework. The recommended growth temperatures for 21 strains were within 3 °C of either 28 °C or 37 °C with an exception for *H. glaciei* DSM15938 and *H. kribbensis* DSM17892, whose recommended growth temperature was 6 °C lower and 5 °C higher than the low reference temperature proposed here, respectively.

Among these 23 strains, twelve strains showed no significant difference (p < 0.01) in either growth parameter (Figure 3; Supplementary Table S6) when their growth environment was adjusted to either one of the proposed reference temperatures. The specific growth rates of *Halomonas salina* DSM5928, *Halomonas kenyensis* DSM17331, and *H. halmophila* DSM5349 significantly improved when cultured at 28 °C, 37 °C and 28 °C, respectively. The specific growth rates of *Halomonas alimentaria* DSM15356 and *Halomonas eurihalina* DSM5720 were significantly reduced when grown at 28 °C, demonstrating clear growth fitness preference for a minimal increase in growth temperature to 30 °C. The maximum attainable optical density reported for *H. elongata* DSM2581, *Halomonas venusta* DSM4743, *Halomonas pacifica* DSM4742, *H. alimentaria* DSM15356, and *H. glaciei* DSM15938 were significantly higher when grown at 28 °C than that in their recommended temperature (p < 0.01). On the other hand, *Halomonas* sp. DSM7328 and *Halomonas mongoliensis* DSM17332 demonstrated a significant decrease in maximum OD_600_ upon minimal change in growth temperature from 30 °C to 28 °C and from 36 °C to 37 °C, respectively. *Halomonas alimentaria* DSM15356 was the only strain demonstrating a significant difference in both specific growth rate and maximum cell density as reported by optical density between the collection-recommended and reference temperatures, with an average growth rate reduction of 41% (µ = 0.221 ± 0.062 h^-1^ vs. µ = 0.131 ± 0.028 h^-1^) and an average maximum cell density increase by 51% (OD_600_ = 2.314 ± 0.289 vs. OD_600_ = 3.494 ± 0.305) when grown at the reference temperature of 28 °C (Supplementary Table S2).

**FIGURE 3:**
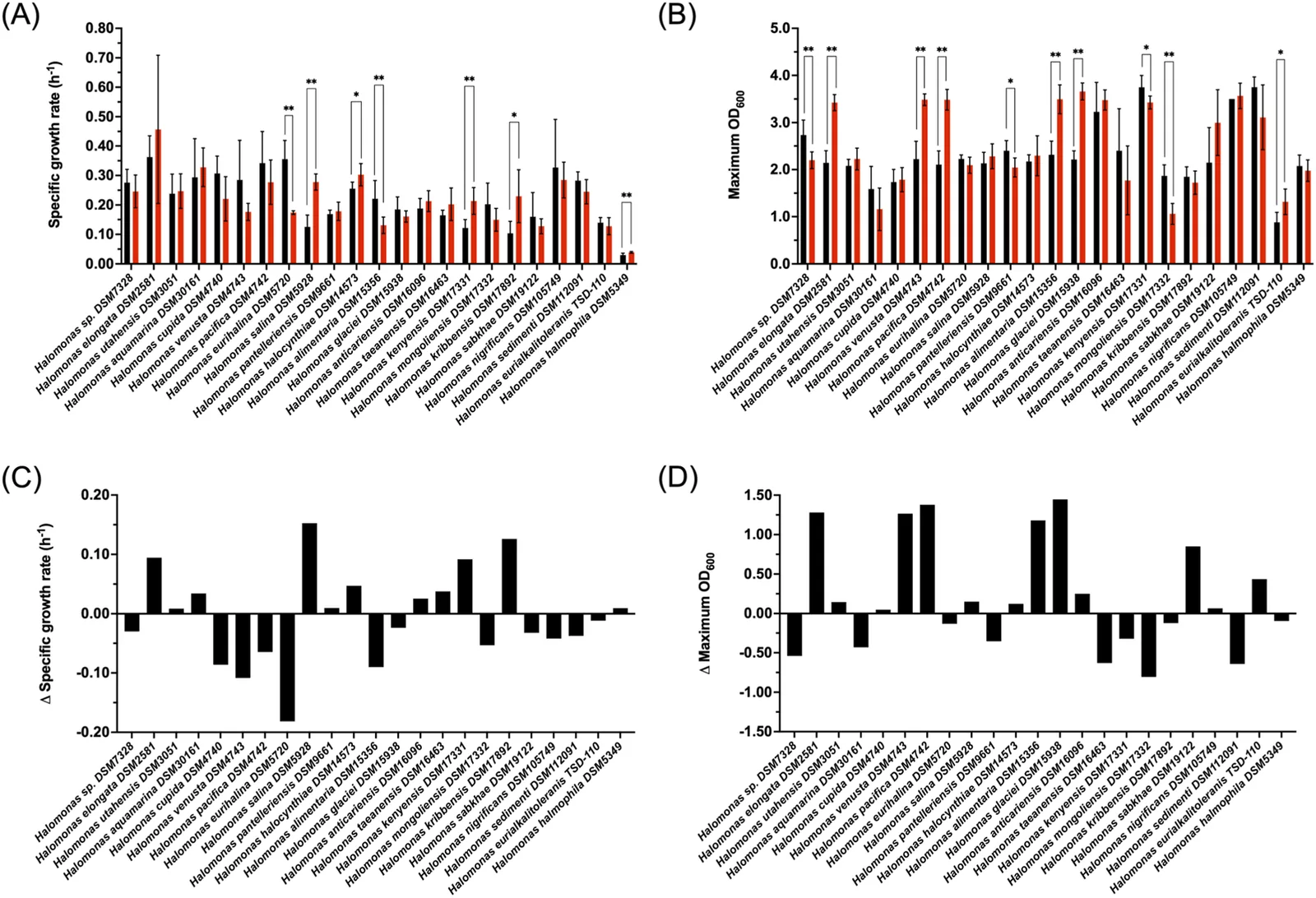
Impact of temperature transition on growth phenotype for 23 Halomonas strains. **(A)** Specific growth rate and **(B)** maximum OD600 were compared between collection-recommended temperatures (black) and the nearest reference temperature of 28 °C or 37 °C (red). Distance plot of **(C)** specific growth rates and **(D)** maximum OD600 displays the difference between recommended and reference temperatures. Positive values of the distance plots indicate superior growth performance at the reference temperature and negative values indicate superior growth performance at the collection-recommended temperature. Error bars represent one standard deviation (n = 6). *p < 0.05; **p < 0.01.

### 3.3 LB broth as a reference growth environment for *Halomonas* cultivation

The strains in this collection were recommended to be cultured in 22 different medium compositions comprising different types of media and varying concentrations of salt, at times, with specific commercial brand requirements for some components to enable growth. Using a standard medium like LB offers major advantages in cost, workflow consistency, and versatility. It simplifies lab management by eliminating the need to inventory and prepare specialised, species-specific recipes. Furthermore, it is the foundation for many routine laboratory protocols. To this end, we explored the suitability of LB as a reference complex medium for supporting *Halomonas* growth. We investigated the growth of the strains in LB supplemented to the recommended salt concentration and grown at the reference temperatures determined from earlier analysis.

Nearly 80% of the strains (64/82) were able to grow in both their recommended medium and in LB. The average specific growth rate of these strains in LB (µ = 0.208 ± 0.136 h^-1^) was comparable, with only 5% difference, to that in their recommended media (µ = 0.220 ± 0.103 h^-1^). Likewise, the maximum optical density attainable by these strains on average in LB (OD_600_ = 2.476 ± 0.986) was similar to that in their recommended medium (OD_600_ = 2.809 ± 0.971), demonstrating a small difference of 12%. *H. campaniensis* DSM15293, *H. venusta* DSM4743, *H. pacifica* DSM4742, *Halomonas sulfidaeris* DSM15722, *Halomonas alkaliphila* DSM16354, *Halomonas taeanensis* DSM16463, *H. kenyensis* DSM17331, *Halomonas sabkhae* DSM19122, *Halomonas johnsoniae* DSM21197, *Halomonas qijiaojingensis* DSM22403, *Halomonas titanicae* DSM22872, *Halomonas songnenensis* DSM25870, and *Halomonas lactosivorans* DSM103220 exhibited no significant difference in either growth parameter when the cultivation medium was changed to LB (p > 0.01). On the other hand, the growth of 13 strains was improved as demonstrated by at least one growth parameter. Both the average specific growth rate and the maximum optical density of *H. mongoliensis* DSM17332, *H. gomseomensis* DSM18042, and *H. halmophila* DSM5349 improved significantly when cultivated in LB (p < 0.01) (Figures 4A,B; Supplementary Table S7). Of the remaining 56 strains, only seven strains demonstrated a significant decrease in both growth parameters.

**FIGURE 4:**
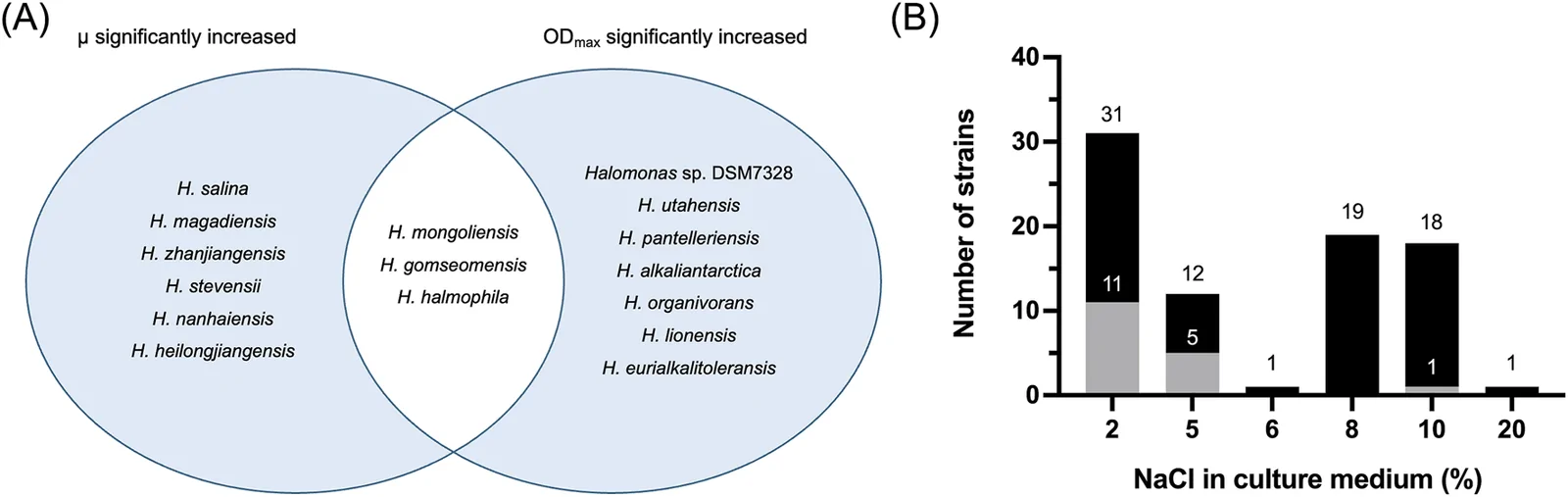
Comparison of growth phenotype between collection-recommended medium and LB medium at matched NaCl concentrations. **(A)** Venn diagram identifying those showing significantly improved specific growth rate and maximum OD600 in LB medium (p < 0.01) among the 64 strains capable of growth in both collection-recommended and LB medium. **(B)** Distribution of strains across NaCl concentrations, showing the total number of strains tested at each concentration (black) alongside those unable to sustain growth in LB medium at the matched concentration (grey).

The growth of *H. janggokensis* DSM18043, the only strain failing to grow in its recommended medium, was recovered in LB supplemented to 10% salt concentration (µ = 0.288 ± 0.106 h^-1^, OD_600_ = 2.455 ± 0.848). LB medium was not able to support the fitness and growth of 17 strains in this collection, demonstrating either no discernible growth or difficulty in growth 2 weeks or more post inoculation indicated by a virtually zero specific growth rate. These strains were primarily those, whose recommended media were supplemented with relatively low salt concentrations of up to 5%, maintaining low to moderate osmolality except one strain with recommended supplementation of 10% NaCl (Figure 4B; Supplementary Table S7). Interestingly, the growth of 75% of these strains could be recovered in LB only by switching to a moderately osmolar environment of 8% salt concentration in the following stage in the framework (Supplementary Table S8). In contrast, only *H. ilicicola* DSM19980, which preferred high osmolality environment (10% salt concentration) was not capable of growth in LB supplemented with 10% salt concentration (Figure 4B; Supplementary Table S7); its growth could be recovered in LB80, by reducing osmolality (Supplementary Table S8) as demonstrated by the next stage in the framework.

### 3.4 Assessing the feasibility of reduced-salt cultivation for *Halomonas*

While elevated salinity is important for supporting *Halomonas* growth, it also poses practical drawbacks, including corrosive effects on laboratory and bioprocessing equipment, making it desirable to minimise salt requirements and dependency wherever possible. To this end, all strains were tested in LB medium at low (2%) and moderate (8%) salt concentrations, denoted as LB20 and LB80, respectively. These were identified above as the two concentrations recommended for the majority of strains in our collection.

The growth of 13 strains, who failed to sustain growth in LB supplemented with their recommended salt concentration, were recovered when cultured either in LB20 or LB80. Only four strains, *H. maura* DSM13445, *H. muralis* DSM14789, *H. kribbensis* DSM17892, and *H. tabrizica* DSM23018, were difficult to culture in LB80 or LB20, despite successfully growing in their respective recommended media, indicating that the marine broth could contain essential components to support their metabolism. The salt requirement of *Halomonas* strains was identified to be sensitive to the choice of medium. A switch from the recommended medium to LB broth resulted in a higher salt requirement for nine strains with preferential growth in LB80 than in LB20.

The growth parameters of 48 strains, which could successfully be cultivated under all tested conditions of salt and choice of medium, were comparatively investigated. The average specific growth rates of these strains when cultivated in LB medium was minimally lower than those cultivated in their recommended media although the difference was not significant (Figure 5A; Supplementary Table S8). A number of strains were determined to grow faster in LB than in their recommended medium demonstrating LB’s growth boosting potential. The expansion of metabolic capabilities enabled by LB was also demonstrated by the expanded 95% quantile, especially towards the upper bound of the growth rate distribution.

**FIGURE 5:**
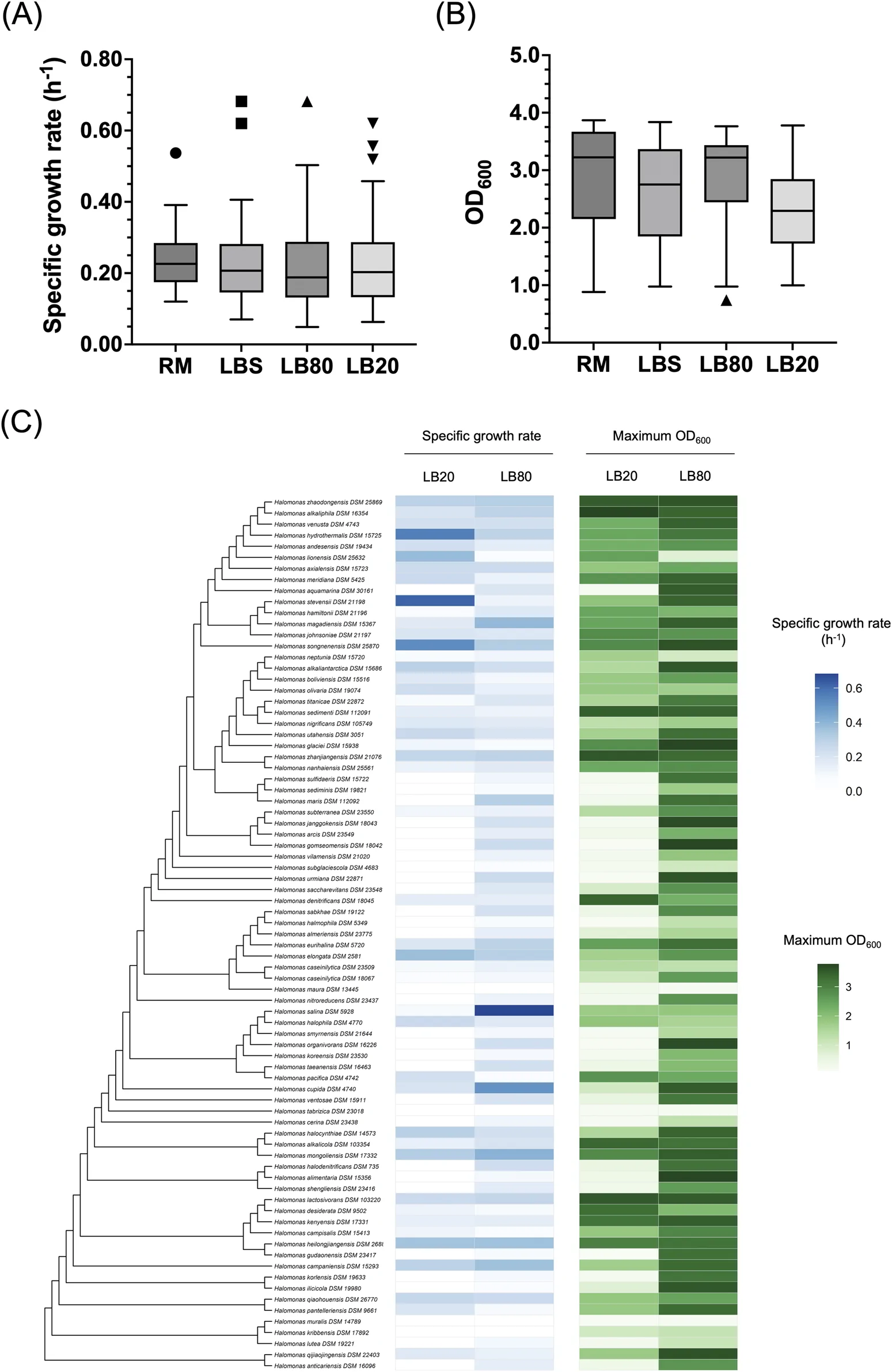
Impact of NaCl concentration on growth characteristics. Tukey box plots displaying **(A)** specific growth rate and **(B)** maximum OD600 across four cultivation conditions of 48 *Halomonas* strains: collection-recommended medium (RM), LB medium at matched NaCl concentration (LBS), LB with 8% NaCl (LB80), and LB with 2% NaCl (LB20). **(C)** Heatmap of growth metrics overlaid against the 16S rRNA-based phylogenetic tree to investigate the relationship between phylogenetic relatedness and growth phenotype of 79 *Halomonas* strains with 16S rRNA data available.

Significant difference was observed in LB80 and LB20 in comparison to RM and LBS. While the average specific growth rates when cultured in LB20 (0.228 ± 0.144 h^-1^) were comparable to those cultured in LB80 (0.218 ± 0.138 h^-1^), there was a noticeable difference between the maximum OD_600_ attained when cultured in LB80 medium in comparison to that in LB20 (OD_600_ = 2.867 ± 0.896 vs. OD_600_ = 2.309 ± 0.858). In agreement with the results presented earlier, the impact of low salt (2%) supplementation was more discernible in LB cultures (Figure 5B; Supplementary Table S8). Elevated osmolality had a greater impact on improving maximum cell densities than on specific growth rates. The maximum OD_600_ of 15 strains was significantly higher in LB80 than in LB20 whereas the converse was true only for two strains, demonstrating that *Halomonas* preferred high osmolality environments for cell density accumulation rather than switching the metabolism towards faster growth.

Growth parameters were overlaid on the phylogenetic tree to visualise their distribution across the collection (Figure 5C). To formally test whether phylogenetic relatedness predicts growth phenotype, entanglement scores, Mantel tests, Blomberg’s K, and Pagel’s λ were calculated across all growth conditions examined (Supplementary Analysis S1, Supplementary Table S10), Specific growth rate showed no significant phylogenetic signal under any condition tested. One exception identified was maximum OD_600_ under low salt conditions (LB20) showed significant phylogenetic signal (Pagel’s λ = 0.757, p < 0.001; Mantel r = 0.061, p = 0.033), indicating that closely related strains tend to reach more similar final cell densities when grown at low salt concentrations than expected by chance. This pattern was not observed as significant under any other conditions for this study and suggests that there may be a phylogenetic constraint on optical density specific to low salt stress rather than a general feature of a growth phenotype in this genus. A visual grouping of strains with low specific growth rates or were unculturable in LB20 were clustered together in the phylogenetic tree, however this pattern did not correspond to a statistically significant signal and should not be interpreted as evidence of evolutionary preference or co-selection. *H. hydrothermalis* DSM15725 and *H. venusta* DSM4743, two closely related strains showed very similar maximum OD_600_ (Δ = 0.141), consistent with the phylogenetic signal for this condition.

The *H. caseinilytica* isolates DSM18067 and DSM23509 were able to grow in both LB20 and LB80 although the maximum optical density attained by the isolates were significantly different in these two media (DSM18067: OD_600_ = 2.603 ± 0.834 in LB80 vs. OD_600_ = 1.055 ± 0.100 in LB20; DSM23509: OD_600_ = 1.278 ± 0.203 in LB80 vs. OD_600_ = 1.429 ± 0.206 in LB20). The 2 *H. lutea* isolates DSM19221 and DSM23508 were only able to grow in LB80 and there was a significant difference in the maximum optical density attained by the two strains (DSM19221: OD_600_ = 1.129 ± 0.387 vs. DSM23508: OD_600_ = 1.787 ± 0.212, both in LB80) (p < 0.01). While these two isolate pairs represent the closest nodes on the phylogenetic tree, the presence of significant differences between them suggests that phylogenetic proximity may be a better proxy for their qualitative phenotypic behaviour than for their quantitative performance.

### 3.5 Towards an LB-based set of reference culturing conditions

Through the stepwise evaluation described above, 27 distinct culture collection-recommended conditions were systematically consolidated into one of four reference conditions LB supplemented to either 2% (LB20) or 8% (LB80) NaCl, cultured at either 28 °C or 37 °C, applicable across 78 *Halomonas* strains examined in this study. The intention of this framework is not to identify a universally optimal condition for all strains, but rather to establish a practical starting point that streamlines preliminary characterisation, synthetic biology protocols, and early-stage bioprocessing workflows across the genus. While the transition to reference conditions improved or impaired the fitness in some strains, other strains showed minimal or dismissible change. *H. kenyensis* DSM17331, *H. johnsoniae* DSM21197, and *H. lactosivorans* DSM103220 exhibited no significant differences in growth characteristics transitioning from their cognate recommended growth medium and osmolality to either LB80 or LB20, demonstrating resilience across nutritional supplementation as well as salt concentrations (Supplementary Tables S7, S8). While the specific growth rate of *H. kenyensis* DSM17331 significantly improved upon transitioning into growth at 37 °C, no other fitness parameters were affected for *H. johnsoniae* DSM21197 and *H. lactosivorans* DSM103220. These remained entirely unaffected throughout the transitioning process and may therefore be considered exceptionally robust strains within the genus.

With the exception of four strains unable to sustain growth in LB, 60 strains were assigned a unique reference condition, either LB20 or LB80, cultivated at 28 °C or 37 °C. The remaining 18 strains were capable of growth under either salt concentration and a reference recommendation was made for temperature alone, selected as whichever of the reference temperatures was closest to the strains original recommended temperature (Figure 6; Supplementary Table 9).

**FIGURE 6:**
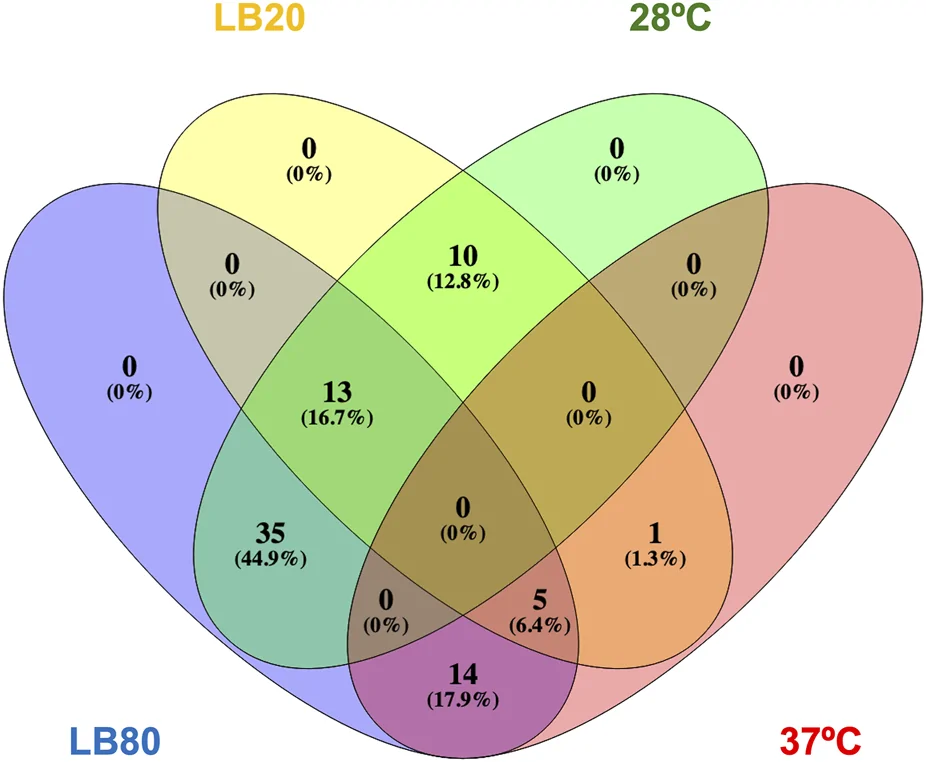
Proposed culture conditions for 82 Halomonas strains. The Venn diagram represents the reference culture conditions indicated by temperature (28 °C and 37 °C) and salt concentrations (2% and 8% NaCl). Six groups of culture conditions were identified. LB80 at 28 °C for 35 strains: *Halomonas sp. DSM7328, Halomonas elongata, H. halodenitrificans, H. utahensis, H. subglaciescola, H. aquamarina, H. cupida, H. venusta, H. eurihalina, H. salina, Halomonas alimentaria, H. alkaliantarctica, H. sulfidaeris, H. ventosae, H. glaciei, H. gudaonensis, H. anticariensis, H. gomseomensis, H. janggokensis, H. caseinilytica* DSM23509, *H. korlensis, H. sediminis, H. ilicicola, H. vilamensis, H. hamiltonii, H. urmiana, H. shengliensis, H. cerina, H. saccharevitans, H. arcis, H. subterranea, H. almeriensis, H. qiaohouensis, H. alkalicola, and H. halmophila*. LB80 at 37 °C for 14 strains: *Halomonas campaniensis, H. magadiensis, H. organivorans, H. taeanensis, H. caseinilytica* DSM18067, *H. sabkhae, H. lutea DSM19221, H. lutea* DSM23508, *H. smyrnensis, H. qijiaojingensis, H. titanicae, H. nitroreducens, H. koreensis, and H. maris*. Either LB80 or LB20 at 28 °C for 13 strains: *H. boliviensis, H. pantelleriensis, H. halocynthiae, H. campisalis, H. axialensis, H. zhanjiangensis, H. johnsoniae, H. stevensii, H. nanhaiensis, H. heilongjiangensis, H. lactosivorans, H. nigrificans, and H. eurialkalitoleransis. LB20 at 28 °C for ten strains: H. halophila, H. pacifica, H. meridiana, H. desiderata, H. neptunia, H. hydrothermalis, H. denitrificans, H. andesensis, H. lionensis, and H. sedimenti. Either LB80 or LB20 at 37 °C for five strains: H. alkaliphila, H. kenyensis, H. mongoliensis, H. zhaodongensis, and H. songnenensis*. LB20 at 37 °C for *H. olivaria*. Four strains, *H. maura, H. muralis, H. kribbensis, and H. tabrizica*, did not grow in either LB80 or LB20 (for Strain ID see Supplementary Table S1).

Growth characteristics were comparatively assessed for the 60 strains assigned a unique reference condition using hierarchical clustering (k = 3, silhouette-verified), applied to both specific growth rate and maximum OD_600_ under collection-recommended (Figure 7A) and proposed reference (Figure 7B) conditions. Clusters were coloured consistently across both panels by rank of mean maximum OD_600_. Under the proposed reference conditions the clusters with the highest OD_600_ (n = 41 and n = 7) did not differ meaningfully from one other while the remaining cluster (n = 12) showed lower maximum OD_600_. Tanglegrams constructed between the 16S rRNA-based phylogenetic tree and growth characteristic clusters revealed no significant correlation under collection recommended conditions and marginal non-significant correlation under reference conditions (Supplementary Figures S3, S4, Supplementary Analysis S1).

**FIGURE 7:**
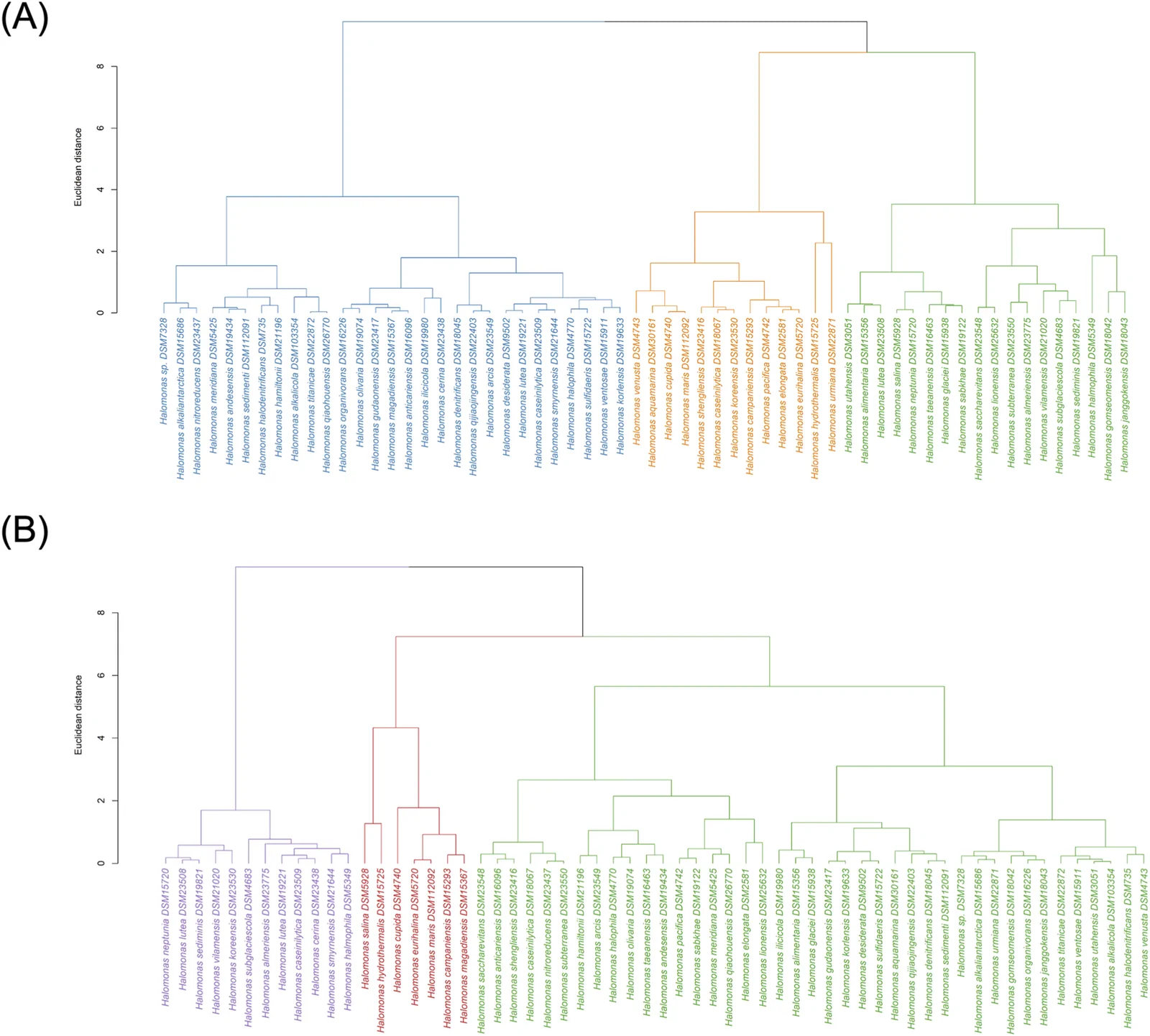
Hierarchical clustering of 60 *Halomonas* strains. Specific growth rates and maximum OD_600_ of 60 *Halomonas* strains were obtained from a cultivation under **(A)** collection-recommended conditions and **(B)** proposed reference conditions, in either low (LB20) or moderate (LB80) salt concentration. Strains were categorised into 3 clusters (k = 3, Ward’s minimum variance method), using both specific growth rate (µ) and maximum OD_600_ (OD_*max*_) jointly. Colour was assigned subsequently: high OD_*max*_ with moderate µ (blue), high OD_*max*_ with high µ (red), moderate OD_*max*_ with high µ (orange), moderate OD_*max*_ with moderate µ (purple), and low OD_*max*_ with low µ (green). There are no strains that were categorised as low OD_*max*_ with moderate or high µ.

To assess scalability of these findings, three strains, *H. magadiensis* DSM15367, *H. stevensii* DSM21198, and *H. elongata* DSM2581, were further cultivated in DASbox® bioreactors under controlled conditions (Figure 8; Supplementary Figures S5). *Halomonas elongata* DSM2581 was included as the type species of the *Halomonas* genus. All strains exhibited higher OD_max_ in LB80 than in LB20 in high-throughput screening. The OD_max_ of all three strains in LB80 bioreactors exceeded those recorded in LB20 bioreactors at both 24 and 49-h marks, mirroring the trend observed at the micro-well plate scale. As in small scale, the OD_max_ of *H. magadiensis* DSM15367 and *H. stevensii* DSM21198 exceeded that of *H. elongata* DSM2581(*H. magadiensis* DSM15367: 4.853 ± 0.075 OD_600_, *H. stevensii* DSM21198: 6.507 ± 0.170 OD_600_, and *H. elongata* DSM2581: 2.943 ± 0.094 OD_600_, Figure 8). These results were encouraging to implicate that the interpretation of the findings in 96-well microplates may not necessarily serve only initial screening purposes, but some properties might, at least partially translate as scale is increased. However, it should be noted that this evaluation was limited to three strains, and endpoint bioreactor measurements were taken, serving as a point of qualitative assessment, and therefore cannot undisputedly show direct comparability or scalability, but merely serve as an encouraging starting point for future evaluations.

**FIGURE 8:**
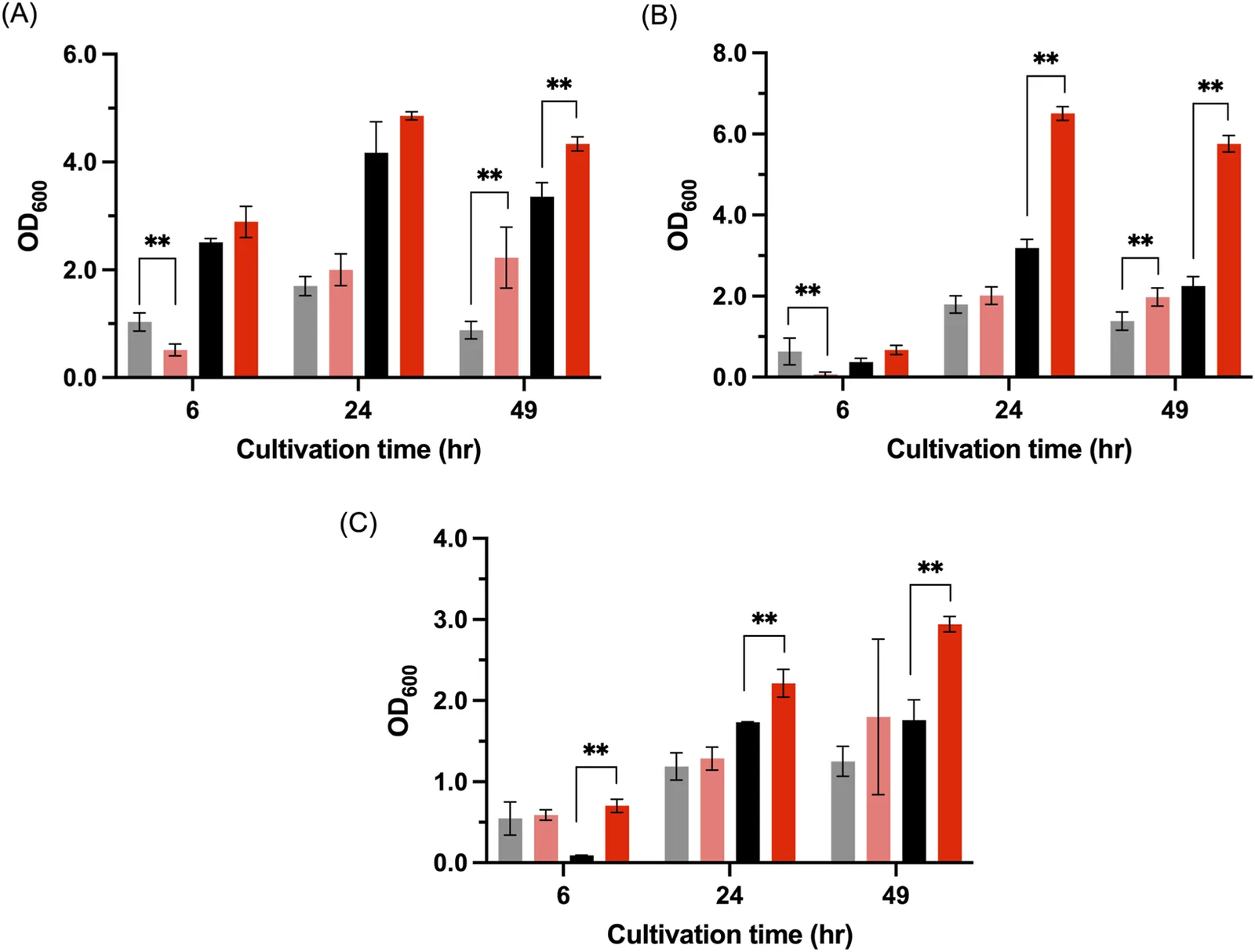
Optical cell densities (OD_600_) of scaled up strains. The maximum growth of **(A)** *H. magadiensis* DSM15367, **(B)** *H. stevensii* DSM21198, and **(C)** *Halomonas elongata* DSM2581 cultures in 96-well plates and DASbox*®* bioreactors under controlled conditions: pH7.0, 30% dissolved oxygen, and temperatures of 28 or 37 °C at 6, 24, and 49 h. The average OD_600_ from 96-well plates (n = 6) and DASbox*®* bioreactors (n = 3) were plotted. The comparison between LB80 and LB20 media was presented; LB20 in 96-well plates (grey), LB80 in 96-well plates (pink), LB20 in bioreactors (black), and LB80 in bioreactors (red).

## 4 Discussion

The *Halomonas* genus is emerging as a promising chassis for industrial biomanufacturing, offering key competitive advantages over conventional microbial platforms. These include the ability to grow at elevated salt concentrations, which substantially reduces contamination risk and energy expenditure associated with sterilisation (Ye and Chen, 2021), and the capacity for open, non-sterile cultivation (Sun et al., 2026), which are properties well-aligned with the sustainability and efficiency demands of next-generation industrial biotechnology. While novel *Halomonas* species continue to be discovered from diverse environments, such as *Halomonas* sp. HK105, recently isolated from a Vietnamese saltern (Nguyen et al., 2026), the absence of agreed, standardised cultivation conditions across the genus remains a significant barrier to streamlining preliminary experimental work and enabling systematic comparison between species.

In this work, an in-house collection of 82 *Halomonas* strains representing 80 species was assembled from the ATCC and DSMZ culture collections. These strains originate from remarkably diverse ecological and anthropogenic environments, ranging from the solid ice layer of Antarctic fast ice in the case of *H. glaciei* DSM15938, to a mural painting in an Austrian chapel in the case of *H. muralis* DSM14789 (Heyrman et al., 2002). This ecological diversity was reflected in the breadth of collection-recommended cultivation conditions, spanning 27 unique condition sets across eight different media, ten cultivation temperatures, and six NaCl concentrations. Our 82 strains were initially characterised under their respective recommended conditions, with all but one strain supporting measurable growth. The exception, *H. janggokensis* DSM18043, displayed a specific growth rate of nearly zero and achieved a maximum OD_600_ of only 0.320 ± 0.130 after 81 h, suggesting that the recommended conditions may be insufficient to support robust growth of this strain.

The scope of this study was deliberately restricted to strains procurable from ATCC and DSMZ, two of the largest culture collections with open global access, using type strains where available. This criterion excludes some of the most extensively studied members of the genus, notably *Halomonas bluephagenesis* (TD01/TD1.0) and *H*. *campaniensis* LS21, neither of which is currently deposited in either collection. This was an intentional trade-off: prioritising strains with guaranteed, reproducible access over exhaustive coverage of the genus, so that any research group could independently procure the same material and test results against the proposed framework. As culture collections continue to expand, strains such as these may become available through ATCC or DSMZ, at which point they would represent valuable additions for benchmarking against the framework presented here.

Preliminary analysis of the collection-recommended cultivation conditions revealed that 72% of the strains were already recommended to grow at either 28 °C or 37 °C. The remaining strains were therefore tested at whichever of these two temperatures was closest to their recommended value, to determine whether this transition could still sustain growth. Growth was confirmed for all strains at either 28 °C or 37 °C, supporting the adoption of these two temperatures as reference cultivation conditions for the in-house *Halomonas* collection.

Identifying a simple, readily accessible medium capable of supporting broad *Halomonas* growth is a practical prerequisite for streamlining cultivation protocols. While MH medium was previously proposed for the phenotypic characterisation of 21 *Halomonas* species (Mata et al., 2002), a simpler alternative was sought for the purposes of this study. Complex media are well-suited to supporting rapid growth, in contrast to minimal media which, while valuable for investigating metabolic profiles, are less appropriate for routine cultivation (Kim and Kim, 2017). In our work, we proposed the comparison across 82 strains (additional 61 strains) with the quantitative growth characterisation and the investigating the relationship between growth parameters and phylogenetic tree. This provided the meaningful and complement with Mata et al. results for selecting the desired *Halomonas* strains and minimising the required tests for both phenotypic and growth characterisation. All strains were tested in LB medium at their reference cultivation temperatures and collection-recommended salt concentrations. Growth was sustained in LB for all but 17 strains, all of which shared Medium 514 as their collection-recommended growth medium. Notably, one of the two type strains of *H*. *lutea* (DSM23508) was among those unable to grow in LB, whereas the other type strain (DSM19221), also recommended for cultivation in Medium 514, was able to sustain growth. Conversely, the growth of *H*. *janggokensis* DSM18043, which had failed to grow in its recommended Medium 830 containing Marine Broth, was recovered in LB. Excluding the strains that failed to grow in LB entirely, and the seven strains that showed a significant decline in both the growth parameters, the remaining 57 strains (approximately 70%) grew adequately in LB, 29 of these strains only showing a decline in one of the two parameters measured. Given the practical cost advantages of a single standardised medium over 27 species-specific formulations, 22 of which are directly related to medium and salt concentration differences, a partial decline in one growth parameter for a subset of strains represents a reasonable trade off against the gains in comparability and laboratory efficiency. The strains displaying specific nutritional requirements showed no clear separation from others on the phylogenetic clustering, suggesting that these phenotypic differences are not readily attributable to 16S rRNA bases phylogenetic relatedness. These observations highlight the complexity of nutrient essentiality within *Halomonas* and reinforce the need for more comprehensive functional and metabolic characterisation of the genus to support its effective biotechnological exploitation.

Elevated NaCl concentrations confer a key advantage for *Halomonas* cultivation by reducing contamination risk in open, non-sterile fermentation environments. However, high salt concentrations also accelerate corrosion of stainless steel, which is the primary material of large-scale bioreactor infrastructure (Yan et al., 2021; Brownlie et al., 2022; Lv et al., 2025), and have been shown to reduce transformation efficiency in molecular biology workflows using LB medium (Coimbra et al., 2025). Identifying salt concentrations that are sufficiently inhibitory to contamination while minimising corrosive burden is therefore of considerable practical importance. Thanakarn et al. proposed 8% NaCl as a moderately low yet contamination-preventive concentration for *Halomonas* sp. DSM7328 cultured in LB (Thanakarn et al., 2026). On this basis, 8% NaCl was evaluated alongside 2% NaCl as candidate reference osmolalities for the cultivation of the in-house *Halomonas* collection.

Transitioning to standardised salt concentrations of either 2% (LB20) or 8% (LB80) recovered growth in 13 strains that had been unable to grow in LB at their collection-recommended salt concentrations. However, four strains, *H. muralis* DSM14789, *H. kribbensis* DSM17892, *H. maura* DSM13445, and *H. tabrizica* DSM23018, could not be recovered under any of the reference conditions tested. The four strains that failed to grow under the simplified LB-based conditions illustrate an expected limitation of a streamlined, genus-wide approach to cultivation. Their lack of growth may reflect specific nutritional or physiological requirements better met by their recommended media; however, determining these requirements would require strain-specific optimisation beyond the scope of the present framework. The aim here is not to replace specialised media or establish optimal conditions for every *Halomonas* strain, but to offer a simple, broadly applicable first cultivation step that can be followed by targeted optimisation where growth is not achieved. Rather than expanding the framework to accommodate a small number of exceptions through additional media components or condition combinations, we therefore preserve its streamlined design and flag these strains as cases warranting further investigation.

Strains generally attained higher maximum cell densities in LB80 than in LB20, consistent with the halophilic properties of the genus, although the magnitude of this response varied considerably across strains, highlighting the importance of strain-level consideration when selecting cultivation conditions in the experiments that follow preliminary analysis. At the other extreme, *H. johnsoniae* DSM21197 and *H. lactosivorans* DSM103220 exhibited no significant differences in any growth characteristic across the full transition, demonstrating exceptional resilience to changes in medium composition, cultivation temperature, and osmolality.

Strain-level differences were observed between the two species examined in this study, *H. lutea* and *H. caseinilytica*, providing interesting insights into their growth characteristics. The 2 *H. lutea* strains exhibited different growth outcomes. *H. lutea* DSM23508 was unable to grow in LB medium at the same salt concentration as that recommended for its cultivation, whereas DSM19221 was able to grow under these conditions. However, both strains were unable to grow in LB20. Although their specific growth rates did not differ significantly in LB80, their maximum OD_600_ values were significantly different (p < 0.01). In contrast, both *H. caseinilytica* strains were able to grow in LB80 and LB20. Their maximum OD_600_ values differed significantly under both conditions, whereas their specific growth rates did not. Collectively, these observations suggest that strain-level variation can influence quantitative growth parameters, particularly maximum OD_600_, while not necessarily affecting qualitative growth ability under the tested conditions.

Following this transition, 60 strains could be assigned a unique reference condition from one of four possibilities: LB20 or LB80 at either 28 °C or 37 °C, while the remaining 18 strains grew without difficulty in LB20 or LB80 at either 28 °C or 37 °C. Hierarchical comparison of phenotypic similarity between recommended and reference conditions indicated a greater convergence under the proposed reference conditions, consistent with the consolidation of cultivation conditions across *Halomonas* strains. Phenotypic similarity showed limited correspondence with 16S rRNA phylogenetic relatedness, revealing no discernible trends with 16S rRNA-based phylogenetic relatedness, indicating that strain-specific cultivation responses within *Halomonas* are not readily predicted by 16S rRNA phylogeny. These findings suggest that additional strain level determinants, including differences in functional genomic content, may contribute to observed variation in the cultivation responses. However, 16S rRNA has well-documented limitations as a marker of overall genomic relatedness (Bartoš et al., 2024), the absence of a clear phylogenetic signal in this study may therefore reflect these limitations rather than genuine absence of evolutionary influence on phenotype. Furthermore, horizontal gene transfer has been documented within *Halomonas* including for genes relevant to osmotic adaptation and polymer metabolism (Cai et al., 2011), providing a potential mechanism by which functional traits may become decoupled from the vertically inherited 16S rRNA signal.

We propose a two-step decision rule for a user approaching an uncharacterised *Halomonas* isolate. As a default, the isolate should be cultivated in LB80, at whichever of the two candidate temperatures (28 °C or 37 °C) is closer to the strain’s original isolation temperature. If no growth is observed under this default condition, LB20 should be tested as a fallback. Applied retrospectively to the 82 strains in this collection, the LB80 default alone accounts for 67 of 82 strains (82%); the LB20 fallback resolves a further 11 strains, bringing cumulative success to 78 of 82 strains (95%). A full step-by-step protocol, including inoculum preparation, plate format, seal, agitation, and criteria for declaring no growth, is provided as Supplementary Text S1.

*Halomonas* strains isolated in future may fall outside of these categories requiring further in-depth investigation based on their unique metabolic requirements. For understanding *Halomonas* phenotypes, we would like to acknowledge the antecedent study by Mata et al. (2002), which provided an important early phenotypic characterisation of *Halomonas* type strains based on a range of functional tests across 21 strains. While our study generated a broader dataset across 82 strains, our objective differs from comprehensive phenotypic characterisation. Rather, we aimed to establish a simple and broadly applicable starting point for culturing members of the *Halomonas* genus, providing common initial conditions that can be applied when working with previously untested strains. These conditions are not intended to represent optimal growth conditions for each strain, but rather a practical baseline from which further optimisation can be undertaken where required. The stepwise streamlining approach used here generated quantitative growth data that enabled us to assess how growth responses changed across the optimisation process and how these responses related to phylogeny. Thus, our findings complement the phenotypic characterisation of Mata et al. (2002) by providing a practical framework for initial cultivation across a broader collection, with the aim of reducing the need for extensive preliminary condition screening when establishing cultivation of *Halomonas* strains.

The reference cultivation framework established in this work represents a significant step towards the systematic and accessible characterisation of the *Halomonas* genus. By consolidating 27 distinct collection-recommended conditions into four standardised reference conditions supporting growth in 95% of the 82 strains examined (Figure 6), this study provides the research community with a practical, resource-efficient entry point for preliminary strain characterisation, comparative physiological studies, and synthetic biology workflows. The demonstrated broad permissiveness of LB medium supplemented with moderate salt concentrations across the genus removes a key practical barrier to wider adoption of *Halomonas* as an industrial chassis, while the identification of strain-specific salt and temperature preferences provides a foundation for more targeted strain selection. The small number of bioreactor runs performed in this evaluation offers an encouraging starting point for future work assessing direct scalability.

The proposed cultivation framework is intended as a simple first-pass approach for recovering *Halomonas* strains rather than as a set of optimal cultivation conditions for every species. Cultivation parameters, such as pH may be an important determinant of growth in some haloalkaliphilic *Halomonas*; regardless, the strains in our collection with reported alkaline growth requirements were successfully cultivated under the conditions evaluated here. We therefore consider that pH does not need to be introduced as an additional variable at this initial stage, as doing so would increase the complexity of the framework without being necessary for initial strain recovery. Once growth has been established, cultivation conditions can of course be further tailored to the physiological requirements of the individual strain, including adjustment of pH where appropriate. Thus, the framework is intended to provide a common and minimally complex starting point, rather than to replace subsequent strain-specific optimisation.

Looking ahead, integration of this framework with functional genomic characterisation will be essential for resolving the strain-specific responses that 16S rRNA-based phylogeny alone cannot predict. Alongside the phenotypic dataset and analytical framework presented in the companion study published in this Special Issue entitled “Elucidation of the growth phenotypes of 82 strains from the *Halomonas* genus”, this work collectively advances the case for *Halomonas* as a tractable, scalable, and sustainable platform for next-generation industrial biotechnology, and offers a transferable model for the standardisation of other understudied and taxonomically diverse microbial genera.

## Data Availability

The datasets generated and analysed in this study can be found in the UCL Data Repository https://doi.org/10.5522/04/32782530.
