# Supplementary material for: Establishing a reference cultivation protocol for systematic characterisation of the Halomonas genus
Source: Front Bioeng Biotechnol. 2026 Sep 24;14:1923905. doi: 10.3389/fbioe.2026.1923905 (PMC13646132; doi:10.3389/fbioe.2026.1923905)
Supplement: Supplementary file 1 [file Supplementaryfile1.docx]

Supplementary Material

for

Establishing a reference cultivation protocol for systematic characterisation of the Halomonas genus

Waritthorn Thanakarn, Alexander Antoine Maria Baumbach, Eva Price, Duygu Dikicioglu^*^

Department of Biochemical Engineering, University College London, London WC1E 6BT, United Kingdom

Supplementary Tables

**Table S1.** 82 *Halomonas* strains procured for this study

| ***Halomonas* species** | **Strain ID** | **Type strain** | **Isolated from** | **Recommended growth temperature (ºC)** | **Recommended medium** | **Recommended [NaCl] (%)** |
| --- | --- | --- | --- | --- | --- | --- |
| *Halomonas* sp. | DSM7328 | No | Israel | 30 | 1428 | 10 |
| *Halomonas elongata* | DSM2581 | Yes | Netherlands | 30 | 514 | 2 |
| *Halomonas campaniensis* | DSM15293 | Yes | Italy | 37 | 1034 | 10 |
| *Halomonas halophila* | DSM4770 | Yes | Unknown | 28 | 514 | 10 |
| *Halomonas boliviensis* | DSM15516 | Yes | Bolivia | 28 | 514 | 8 |
| *Halomonas halodenitrificans* | DSM735 | Yes | Canada | 28 | 514 | 8 |
| *Halomonas utahensis* | DSM3051 | Yes | USA | 30 | 514 | 8 |
| *Halomonas subglaciescola* | DSM4683 | Yes | Antarctica | 28 | 514 | 8 |
| *Halomonas aquamarina* | DSM30161 | Yes | Unknown | 25 | 514 | 2 |
| *Halomonas cupida* | DSM4740 | Yes | Unknown | 25 | 514 | 2 |
| *Halomonas venusta* | DSM4743 | Yes | Unknown | 25 | 514 | 2 |
| *Halomonas pacifica* | DSM4742 | Yes | Unknown | 26 | 514 | 2 |
| *Halomonas meridiana* | DSM5425 | Yes | Antarctica | 28 | 514 | 2 |
| *Halomonas eurihalina* | DSM5720 | Yes | Spain | 30 | 1689 | 6.2 |
| *Halomonas salina* | DSM5928 | Yes | Spain | 30 | 514 | 8 |
| *Halomonas desiderata* | DSM9502 | Yes | Germany | 28 | 514 | 2 |
| *Halomonas pantelleriensis* | DSM9661 | Yes | Italy | 30 | 2049 | 10.2 |
| *Halomonas maura* | DSM13445 | Yes | Morocco | 28 | 514 | 5 |
| *Halomonas halocynthiae* | DSM14573 | Yes | Japan | 25 | 514 | 2 |
| *Halomonas muralis* | DSM14789 | Yes | Austria | 28 | 514 | 2 |
| *Halomonas alimentaria* | DSM15356 | Yes | South Korea | 30 | 514 | 2 |
| *Halomonas magadiensis* | DSM15367 | Yes | Kenya | 37 | 514 | 8 |
| *Halomonas campisalis* | DSM15413 | Yes | USA | 28 | 514 | 2 |
| *Halomonas alkaliantarctica* | DSM15686 | No | Antarctica | 28 | 514 | 10 |
| *Halomonas neptunia* | DSM15720 | Yes | Canada | 28 | 514 | 8 |
| *Halomonas sulfidaeris* | DSM15722 | Yes | Canada | 28 | 514 | 8 |
| *Halomonas axialensis* | DSM15723 | Yes | Pacific Ocean | 28 | 514 | 8 |
| *Halomonas hydrothermalis* | DSM15725 | Yes | Unknown | 28 | 514 | 8 |
| *Halomonas ventosae* | DSM15911 | Yes | Spain | 28 | 514 | 5 |
| *Halomonas glaciei* | DSM15938 | Yes (proposed) | Antarctica | 22 | 514 | 2 |
| *Halomonas gudaonensis* | DSM23417 | Yes | China | 28 | 514 | 2 |
| *Halomonas anticariensis* | DSM16096 | Yes | Spain | 30 | 514 | 4.5 |
| *Halomonas organivorans* | DSM16226 | Yes | Spain | 37 | 514 | 8 |
| *Halomonas alkaliphila* | DSM16354 | Yes | Italy | 37 | 514 | 8 |
| *Halomonas taeanensis* | DSM16463 | Yes | South Korea | 35 | 514 | 10 |
| *Halomonas kenyensis* | DSM17331 | Yes | Africa | 36 | 514 | 8 |
| *Halomonas mongoliensis* | DSM17332 | Yes | Mongolia | 36 | 514 | 8 |
| *Halomonas kribbensis* | DSM17892 | Yes | Republic of Korea | 33 | 514 | 5 |
| *Halomonas gomseomensis* | DSM18042 | Yes | South Korea | 28 | 830 | 10 |
| *Halomonas janggokensis* | DSM18043 | Yes | South Korea | 28 | 830 | 10 |
| *Halomonas denitrificans* | DSM18045 | Yes | South Korea | 28 | 514 | 10 |
| *Halomonas caseinilytica* | DSM18067 | No | Egypt | 37 | 372 | 20.2 |
| *Halomonas caseinilytica* | DSM23509 | Yes | China | 28 | 514 | 5 |
| *Halomonas olivaria* | DSM19074 | Yes | Morocco | 37 | 514 | 8 |
| *Halomonas sabkhae* | DSM19122 | Yes | Algeria | 40 | 514 | 10 |
| *Halomonas lutea* | DSM19221 | Yes | China | 37 | 514 | 10 |
| *Halomonas lutea* | DSM23508 | Yes | China | 37 | 514 | 5 |
| *Halomonas andesensis* | DSM19434 | Yes | Bolivia | 28 | 514 | 2 |
| *Halomonas korlensis* | DSM19633 | Yes | China | 28 | 514 | 10 |
| *Halomonas sediminis* | DSM19821 | Yes | China | 37 | 514 | 10 |
| *Halomonas ilicicola* | DSM19980 | Yes | Spain | 28 | 514 | 10 |
| *Halomonas vilamensis* | DSM21020 | Yes | Argentina | 28 | 514 | 10 |
| *Halomonas zhanjiangensis* | DSM21076 | Yes | China | 28 | 514 | 5 |
| *Halomonas hamiltonii* | DSM21196 | Yes | USA | 28 | 514 | 2 |
| *Halomonas johnsoniae* | DSM21197 | Yes | USA | 28 | 514 | 2 |
| *Halomonas stevensii* | DSM21198 | Yes | USA | 28 | 514 | 2 |
| *Halomonas smyrnensis* | DSM21644 | Yes | Turkey | 37 | 514 | 10 |
| *Halomonas qijiaojingensis* | DSM22403 | Yes | China | 37 | 514 | 7 |
| *Halomonas urmiana* | DSM22871 | Yes | Iran | 28 | 514 | 2 |
| *Halomonas titanicae* | DSM22872 | Yes | Canada | 37 | 514 | 8 |
| *Halomonas tabrizica* | DSM23018 | Yes | Iran | 28 | 514 | 2 |
| *Halomonas shengliensis* | DSM23416 | Yes | China | 28 | 514 | 2 |
| *Halomonas nitroreducens* | DSM23437 | Yes | Chile | 37 | 514 | 5 |
| *Halomonas cerina* | DSM23438 | Yes | Spain | 28 | 514 | 8 |
| *Halomonas koreensis* | DSM23530 | Yes | South Korea | 37 | 514 | 2 |
| *Halomonas saccharevitans* | DSM23548 | Yes | China | 28 | 514 | 2 |
| *Halomonas arcis* | DSM23549 | Yes | China | 28 | 514 | 2 |
| *Halomonas subterranea* | DSM23550 | Yes | China | 28 | 514 | 2 |
| *Halomonas almeriensis* | DSM23775 | Yes | Spain | 28 | 514 | 5 |
| *Halomonas nanhaiensis* | DSM25561 | Yes | China | 28 | 514 | 5 |
| *Halomonas lionensis* | DSM25632 | Yes (Proposed) | France | 28 | 514 | 2 |
| *Halomonas zhaodongensis* | DSM25869 | Yes (Proposed) | China | 37 | 514 | 7 |
| *Halomonas songnenensis* | DSM25870 | Yes | China | 37 | 514 | 5 |
| *Halomonas qiaohouensis* | DSM26770 | Yes | China | 28 | 514 | 2 |
| *Halomonas heilongjiangensis* | DSM26881 | Yes | China | 28 | 514 | 5 |
| *Halomonas lactosivorans* | DSM103220 | Yes | China | 28 | 514 | 2 |
| *Halomonas alkalicola* | DSM103354 | Yes | China | 28 | 514 | 2 |
| *Halomonas nigrificans* | DSM105749 | Yes | Germany | 30 | 514 | 2 |
| *Halomonas sedimenti* | DSM112091 | Yes | Indian Ocean | 30 | 514 | 2 |
| *Halomonas maris* | DSM112092 | Yes | Indian Ocean | 37 | 514 | 2 |
| *Halomonas eurialkalitoleransis* | ATCC TSD-110 | Yes | USA | 30 | TSD-110 Medium | 10 |
| *Halomonas halmophila* | DSM5349 | Yes | Unknown | 25 | 514 | 10 |

**Table S2.** Recommended culture media compositions

| **Medium** | **Components** | **Amount (per litre)** |
| --- | --- | --- |
| Marine broth 2216  (Millipore, Cat. no. 76448) | Peptone | 5 g |
|  | Yeast extract | 1 g |
|  | Ferric citrate | 0.1 g |
|  | Sodium chloride | 19.45 g |
|  | Magnesium chloride | 8.8 g ^a^ |
|  | Sodium sulfate ^a^ | 3.24 g |
|  | Calcium chloride | 1.8 g |
|  | Potassium chloride | 0.55 g |
|  | Sodium bicarbonate | 0.16 g |
|  | Potassium bromide | 0.08 g |
|  | Strontium chloride | 34 mg |
|  | Boric acid | 22 mg |
|  | Sodium silicate | 4 mg |
|  | Sodium fluoride | 2.4 mg |
|  | Ammonium nitrate | 1.6 mg |
|  | Disodium phosphate | 8 mg |
| Difco^TM^ Marine broth 2216  (BD, Cat. no. 279110) | Peptone | 5 g |
|  | Yeast extract | 1 g |
|  | Ferric citrate | 0.1 g |
|  | Sodium chloride | 19.45 g |
|  | Magnesium chloride | 5.9 g ^a^ |
|  | Magnesium sulfate ^a^ | 3.24 g |
|  | Calcium chloride | 1.8 g |
|  | Potassium chloride | 0.55 g |
|  | Sodium bicarbonate | 0.16 g |
|  | Potassium bromide | 0.08 g |
|  | Strontium chloride | 34 mg |
|  | Boric acid | 22 mg |
|  | Sodium silicate | 4 mg |
|  | Sodium fluoride | 2.4 mg |
|  | Ammonium nitrate | 1.6 mg |
|  | Disodium phosphate | 8 mg |
| Medium 1034 ALKALIPHILIC HALOMONAS MEDIUM | Yeast extract (Difco Gibco Cat. no. 210929) | 10 g |
|  | Sodium citrate | 3 g |
|  | Sodium chloride | 100 g |
|  | MgSO_4_·7H_2_O | 1 g |
|  | Na_2_CO_3_ | 3 g |
|  | Solution A | 1 mL |
|  | Solution B | 1 mL |
|  | Solution A: | |
|  | MnCl_2_·4H_2_O | 0.36 g |
|  | Distilled water | 1000 mL |
|  | Solution B: | |
|  | FeSO_4_·7H_2_O | 50 g |
|  | Distilled water | 1000 mL |
|  | Adjust to pH 9 with 3M NaOH | |
| Medium 1689 MH medium (ATCC) | Yeast extract | 10 g |
|  | Peptone No.3 | 5 g |
|  | Glucose | 1 g |
|  | Sodium chloride | 60.7 g |
|  | MgCl_2_·6H_2_O | 15 g |
|  | MgSO_4_·7H_2_O | 7.4 g |
|  | CaCl_2_ | 0.27 g |
|  | KCl | 1.5 g |
|  | NaHCO_3_ | 45 mg |
|  | NaBr | 19 mg |
| Medium 2049 Haloalkalophile medium (ATCC) | Solution A: | |
|  | Na_2_CO_3_ | 3 g |
|  | Sodium chloride | 100 g |
|  | Solution B: | |
|  | KCl | 2 g |
|  | MgSO_4_·7H_2_O | 1 g |
|  | Yeast extract | 10 g |
|  | Glucose | 1 g |
|  | MnCl_2_·4H_2_O | 3.6 mg |
|  | FeSO_4_·7H_2_O | 5 mg |
|  | Adjust to pH 9 with 3M NaOH | |
| Medium 830 R2A medium (+ 10%NaCl) | Yeast extract | 0.5 g |
|  | Peptone (Difco No.3) | 0.5 g |
|  | Casamino acids | 0.5 g |
|  | Glucose | 0.5 g |
|  | Starch (soluble) | 0.5 g |
|  | Na-pyruvate | 0.3 g |
|  | K_2_HPO_4_ | 0.3 g |
|  | MgSO_4_·7H_2_O | 0.05 g |
|  | Sodium chloride | 100 g |
| Medium 372 Halobacteria Medium | Yeast extract (Fisher BP1422) | 5 g |
|  | Casamino acids | 5 g |
|  | Na-glutamate | 1 g |
|  | KCl | 2 g |
|  | Na_3_-citrate | 3 g |
|  | MgSO_4_·7H_2_O | 20 g |
|  | NaCl | 200 g |
|  | FeCl_2_·4H_2_O | 36 mg |
|  | MnCl_2_·4H_2_O | 0.36 mg |
| Medium for TSD-110 ATCC | Difco nutrient broth base (BD Cat. no. 23400) | 8 g |
|  | Sodium chloride | 100 g |
|  | Glucose | 1% w/v |
|  | Adjust to pH 9 | |

^a^ denotes the difference in medium composition between the Marine Broth supplied by Merck Millipore and BD Difco.

**Table S3.** 16S rRNA database of 79 *Halomonas* spp. for generating phylogenetic tree

| ***Halomonas* species** | **Strain ID.** | **Database** | **Accession number** |
| --- | --- | --- | --- |
| *Halomonas alimentaria* | DSM15356 | GenBank | AF211860.1 |
| *Halomonas alkaliantarctica* | DSM15686 | GenBank | AJ564880.1 |
| *Halomonas alkalicola* | DSM103354 | GenBank | KU530128.1 |
| *Halomonas alkaliphila* | DSM16354 | GenBank | AJ640133.1 |
| *Halomonas almeriensis* | DSM23775 | GenBank | AY858696.1 |
| *Halomonas andesensis* | DSM19434 | GenBank | EF622233.2 |
| *Halomonas anticariensis* | DSM16096 | GenBank | KY034384 |
| *Halomonas aquamarina* | DSM30161 | GenBank | AJ306888.1 |
| *Halomonas arcis* | DSM23549 | GenBank | EF144147.1 |
| *Halomonas axialensis* | DSM15723 | GenBank | AF212206 |
| *Halomonas boliviensis* | DSM15516 | GenBank | AY245449 |
| *Halomonas campaniensis* | DSM15293 | GenBank | AJ515365.2 |
| *Halomonas campisalis* | DSM15413 | GenBank | AF054286.1 |
| *Halomonas caseinilytica* | DSM23509 | GenBank | EF527874.1 |
| *Halomonas caseinilytica* | DSM18067 | GenBank | AM238662.1 |
| *Halomonas cerina* | DSM23438 | GenBank | EF613112.1 |
| *Halomonas cupida* | DSM4740 | GenBank | L42615.1 |
| *Halomonas denitrificans* | DSM18045 | GenBank | AM229317 |
| *Halomonas desiderata* | DSM9502 | GenBank | X92417 |
| *Halomonas elongata* | DSM2581 | GenBank | FN869568.2 |
| *Halomonas eurialkalitoleranis* | ATTC TSD-110 | Not available | Not available |
| *Halomonas eurihalina* | DSM5720 | GenBank | L42620.1, X87218.1 |
| *Halomonas glaciei* | DSM15938 | GenBank | AJ431369.1 |
| *Halomonas gomseomensis* | DSM18042 | GenBank | AM229314 |
| *Halomonas gudaonensis* | DSM23417 | GenBank | DQ421808.1 |
| *Halomonas halmophila* | DSM5349 | GenBank | AJ306889 |
| *Halomonas halocynthiae* | DSM14573 | GenBank | AJ417388.1 |
| *Halomonas halodenitrificans* | DSM735 | GenBank | L04942.1 |
| *Halomonas halophila* | DSM4770 | GenBank | AB681879 |
| *Halomonas hamiltonii* | DSM21196 | GenBank | AM941396.2 |
| *Halomonas heilongjiangensis* | DSM26881 | GenBank | KF479230.1 |
| *Halomonas hydrothermalis* | DSM15725 | GenBank | AF212218 |
| *Halomonas ilicicola* | DSM19980 | GenBank | EU218533.1 |
| *Halomonas janggokensis* | DSM18043 | GenBank | AM229315 |
| *Halomonas johnsoniae* | DSM21197 | GenBank | AM941399.2 |
| *Halomonas kenyensis* | DSM17331 | GenBank | AY962237.1 |
| *Halomonas koreensis* | DSM23530 | GenBank | AY382579.2 |
| *Halomonas korlensis* | DSM19633 | GenBank | EU085033.1 |
| *Halomonas kribbensis* | DSM17892 | GenBank | DQ280368.1 |
| *Halomonas lactosivorans* | DSM103220 | GenBank | KY039330 |
| *Halomonas lionensis* | DSM25632 | GenBank | NR_126205.1 |
| *Halomonas. lutea* | DSM19221, DSM23508 | GenBank | EF674852.1 |
| *Halomonas magadiensis* | DSM15367 | GenBank | X92150.1 |
| *Halomonas maris* | DSM112092 | GenBank | MT372903.1 |
| *Halomonas maura* | DSM13445 | GenBank | AJ271864 |
| *Halomonas meridiana* | DSM5425 | GenBank | AJ306891.1 |
| *Halomonas mongoliensis* | DSM17332 | GenBank | AY962236.1 |
| *Halomonas muralis* | DSM14789 | GenBank | AJ320530.1 |
| *Halomonas nanhaiensis* | DSM25561 | GenBank | JX870002.1 |
| *Halomonas neptunia* | DSM15720 | GenBank | AF212202.2 |
| *Halomonas nigrificans* | DSM105749 | GenBank | MG030686.1 |
| *Halomonas nitroreducens* | DSM23437 | GenBank | EF613113.1 |
| *Halomonas olivaria* | DSM19074 | GenBank | DQ645593.1 |
| *Halomonas organivorans* | DSM16226 | GenBank | AJ616910.1 |
| *Halomonas pacifica* | DSM4742 | GenBank | AB681734.1 |
| *Halomonas pantelleriensis* | DSM9661 | GenBank | X93493.1 |
| *Halomonas qiaohouensis* | DSM26770 | GenBank | KC237714.1 |
| *Halomonas qijiaojingensis* | DSM22403 | GenBank | HQ832735.1 |
| *Halomonas sabkhae* | DSM19122 | GenBank | EF442769.1 |
| *Halomonas saccharevitans* | DSM23548 | GenBank | EF144149.1 |
| *Halomonas salina* | DSM5928 | GenBank | AJ295145 |
| *Halomonas sedimenti* | DSM112091 | GenBank | MT372904.1 |
| *Halomonas sediminis* | DSM19821 | GenBank | EU135707.1 |
| *Halomonas shengliensis* | DSM23416 | GenBank | EF121853.1 |
| *Halomonas smyrnensis* | DSM21644 | GenBank | DQ131909.2 |
| *Halomonas songnenensis* | DSM25870 | GenBank | JQ762289.2 |
| *Halomonas stevensii* | DSM21198 | GenBank | AM941388.3 |
| *Halomonas subglaciescola* | DSM4683 | GenBank | AJ306892.1 |
| *Halomonas subterranea* | DSM23550 | GenBank | EF144148.1 |
| *Halomonas sulfidaeris* | DSM15722 | GenBank | AF212204.2 |
| *Halomonas tabrizica* | DSM23018 | GenBank | EU305728.1 |
| *Halomonas taeanensis* | DSM16463 | GenBank | AY671975.1 |
| *Halomonas titanicae* | DSM22872 | GenBank | FN433898.1, GQ354374.1 |
| *Halomonas urmiana* | DSM22871 | GenBank | EU305729.1 |
| *Halomonas utahensis* | DSM3051 | GenBank | NR_042068.1 |
| *Halomonas ventosae* | DSM15911 | GenBank | AY268080.1 |
| *Halomonas venusta* | DSM4743 | GenBank | AJ306894.1 |
| *Halomonas vilamensis* | DSM21020 | GenBank | EU557315.1 |
| *Halomonas zhanjiangensis* | DSM21076 | GenBank | FJ429198.1 |
| *Halomonas zhaodongensis* | DSM25869 | GenBank | JQ762286.2 |
| *Halomonas sp.* | DSM7328 | Not available | Not available |

Table S4. R packages and bioinformatics tools used in this study

| **Package** | **Version** | **Reference*** |
| --- | --- | --- |
| *ape* | 5.8.1 | (Paradis and Schliep, 2019) |
| *cluster* | 2.1.8.2 | (Maechler et al., 2026) |
| *dendextend* | 1.19.1 | (Galili, 2015) |
| *dplyr* | 1.2.0 | (Wickham et al., 2026) |
| *fields* | 17.3.0 | (Douglas Nychka et al., 2021) |
| *ggnewscale* | 0.5.2 | (Campitelli, n.d.) |
| *ggplot2* | 4.0.3 | (Wickham, 2016) |
| *ggtree* | 3.16.3 | (Yu et al., 2017) |
| *gplots* | 3.3.0 | (Warnes et al., 2025) |
| IQ-TREE | 3.0.1 | (Wong et al., 2026) |
| MAFFT | 7.0.0 | (Katoh and Standley, 2013) |
| *phangorn* | 2.12.1 | (Schliep, 2011) |
| *phytools* | 2.5.2 | (Revell, 2012, 2024) |
| *plotly* | 4.12.0 | (Sievert, 2020) |
| *treeio* | 1.32.0 | (Wang et al., 2020) |
| *trimAI* | 1.5.0 | (Capella-Gutiérrez et al., 2009) |
| *vegan* | 2.7.5 | (Oksanen et al., 2022) |
| *viridis* | 0.6.5 | (Garnier et al., 2024) |

***** Campitelli, E. (n.d.). *ggnewscale: Multiple Fill and Colour Scales in “ggplot2.”* doi: 10.5281/zenodo.2543762

Capella-Gutiérrez, S., Silla-Martínez, J. M., and Gabaldón, T. (2009). trimAl: a tool for automated alignment trimming in large-scale phylogenetic analyses. *Bioinformatics* 25, 1972–1973. doi: 10.1093/bioinformatics/btp348

Douglas Nychka, Reinhard Furrer, John Paige, and Stephan Sain (2021). fields: Tools for spatial data. Available at: https://github.com/dnychka/fieldsRPackage

Galili, T. (2015). dendextend: an R package for visualizing, adjusting and comparing trees of hierarchical clustering. *Bioinformatics* 31, 3718–3720. doi: 10.1093/bioinformatics/btv428

Garnier, Simon, Ross, Noam, Rudis, Robert, et al. (2024). *viridis(Lite) - Colorblind-Friendly Color Maps for R*. doi: 10.5281/zenodo.4679423

Katoh, K., and Standley, D. M. (2013). MAFFT Multiple Sequence Alignment Software Version 7: Improvements in Performance and Usability. *Molecular Biology and Evolution* 30, 772–780. doi: 10.1093/molbev/mst010

Maechler, M., Rousseeuw, P., Struyf, A., Hubert, M., and Hornik, K. (2026). *cluster: Cluster Analysis Basics and Extensions*. Available at: https://CRAN.R-project.org/package=cluster

Oksanen, J., Simpson, G., Blanchet, F. G., Kindt, R., Legendre, P., Minchin, P., et al. (2022). *vegan community ecology package version 2.6-2 April 2022*.

Paradis, E., and Schliep, K. (2019). ape 5.0: an environment for modern phylogenetics and evolutionary analyses in R. *Bioinformatics* 35, 526–528. doi: 10.1093/bioinformatics/bty633

Revell, L. (2024). phytools 2.0: an updated R ecosystem for phylogenetic comparative methods (and other things). *PeerJ* 12, e16505. doi: 10.7717/peerj.16505

Revell, L. J. (2012). phytools: an R package for phylogenetic comparative biology (and other things). *Methods in Ecology and Evolution* 3, 217–223. doi: https://doi.org/10.1111/j.2041-210X.2011.00169.x

Schliep, K. P. (2011). phangorn: phylogenetic analysis in R. *Bioinformatics* 27, 592–593. doi: 10.1093/bioinformatics/btq706

Sievert, C. (2020). *Interactive Web-Based Data Visualization with R, plotly, and shiny*. Chapman and Hall/CRC. Available at: https://plotly-r.com

Wang, L.-G., Lam, T. T.-Y., Xu, S., Dai, Z., Zhou, L., Feng, T., et al. (2020). Treeio: An R Package for Phylogenetic Tree Input and Output with Richly Annotated and Associated Data. *Molecular Biology and Evolution* 37, 599–603. doi: 10.1093/molbev/msz240

Warnes, G. R., Bolker, B., Bonebakker, L., Gentleman, R., Huber, W., Liaw, A., et al. (2025). *gplots: Various R Programming Tools for Plotting Data*. Available at: https://github.com/talgalili/gplots

Wickham, H. (2016). *ggplot2: Elegant Graphics for Data Analysis*. Springer-Verlag New York. Available at: https://ggplot2.tidyverse.org

Wickham, H., François, R., Henry, L., Müller, K., and Vaughan, D. (2026). *dplyr: A Grammar of Data Manipulation*. Available at: https://github.com/tidyverse/dplyr

Wong, T. K. F., Ly-Trong, N., Ren, H., Demotte, P., Baños, H., Roger, A. J., et al. (2026). IQ-TREE 3: phylogenomic inference software using complex evolutionary models. *Molecular Biology and Evolution* 43, msag117. doi: 10.1093/molbev/msag117

Yu, G., Smith, D., Zhu, H., Guan, Y., and Lam, T. T.-Y. (2017). ggtree: an R package for visualization and annotation of phylogenetic trees with their covariates and other associated data. *Methods in Ecology and Evolution* 8, 28–36. doi: 10.1111/2041-210X.12628

**Table S5.** Mean specific growth rate (μ**)** and maximum OD_600_ of 82 *Halomonas* strains in their respectively recommended culturing conditions (n = 6, with standard deviation)

| ***Halomonas* species** | **Strain ID** | **Recommended growth temperature (ºC)** | **Recommended medium** | **Recommended [NaCl] (%)** | **μ (h^-1^)** | | **Maximum OD_600_** | |
| --- | --- | --- | --- | --- | --- | --- | --- | --- |
|  |  |  |  |  | **Average** | **Standard Deviation** | **Average** | **Standard Deviation** |
| *Halomonas sp.* | DSM7328 | 30 | 1428 | 10 | 0.276 | 0.045 | 2.738 | 0.314 |
| *Halomonas elongata* | DSM2581 | 30 | 514 | 2 | 0.362 | 0.073 | 2.144 | 0.256 |
| *Halomonas campaniensis* | DSM15293 | 37 | 1034 | 10 | 0.388 | 0.045 | 2.232 | 0.668 |
| *Halomonas halophila* | DSM4770 | 28 | 514 | 10 | 0.201 | 0.063 | 3.445 | 0.279 |
| *Halomonas boliviensis* | DSM15516 | 28 | 514 | 8 | 0.297 | 0.082 | 2.037 | 0.233 |
| *Halomonas halodenitrificans* | DSM735 | 28 | 514 | 8 | 0.317 | 0.039 | 3.551 | 0.141 |
| *Halomonas utahensis* | DSM3051 | 30 | 514 | 8 | 0.238 | 0.066 | 2.079 | 0.138 |
| *Halomonas subglaciescola* | DSM4683 | 28 | 514 | 8 | 0.109 | 0.022 | 1.344 | 0.715 |
| *Halomonas aquamarina* | DSM30161 | 25 | 514 | 2 | 0.294 | 0.131 | 1.588 | 0.478 |
| *Halomonas cupida* | DSM4740 | 25 | 514 | 2 | 0.307 | 0.059 | 1.737 | 0.265 |
| *Halomonas venusta* | DSM4743 | 25 | 514 | 2 | 0.285 | 0.135 | 2.221 | 0.377 |
| *Halomonas pacifica* | DSM4742 | 26 | 514 | 2 | 0.342 | 0.108 | 2.106 | 0.289 |
| *Halomonas meridiana* | DSM5425 | 28 | 514 | 2 | 0.274 | 0.034 | 3.869 | 0.202 |
| *Halomonas eurihalina* | DSM5720 | 30 | 1689 | 6.2 | 0.355 | 0.063 | 2.225 | 0.085 |
| *Halomonas salina* | DSM5928 | 30 | 514 | 8 | 0.125 | 0.040 | 2.132 | 0.234 |
| *Halomonas desiderata* | DSM9502 | 28 | 514 | 2 | 0.176 | 0.023 | 3.757 | 0.297 |
| *Halomonas pantelleriensis* | DSM9661 | 30 | 2049 | 10.2 | 0.168 | 0.014 | 2.398 | 0.216 |
| *Halomonas maura* | DSM13445 | 28 | 514 | 5 | 0.054 | 0.019 | 2.819 | 0.817 |
| *Halomonas halocynthiae* | DSM14573 | 25 | 514 | 2 | 0.256 | 0.022 | 2.173 | 0.140 |
| *Halomonas muralis* | DSM14789 | 28 | 514 | 2 | 0.226 | 0.075 | 0.983 | 0.136 |
| *Halomonas alimentaria* | DSM15356 | 30 | 514 | 2 | 0.221 | 0.062 | 2.314 | 0.289 |
| *Halomonas magadiensis* | DSM15367 | 37 | 514 | 8 | 0.181 | 0.016 | 3.187 | 0.167 |
| *Halomonas campisalis* | DSM15413 | 28 | 514 | 2 | 0.179 | 0.024 | 3.827 | 0.166 |
| *Halomonas alkaliantarctica* | DSM15686 | 28 | 514 | 10 | 0.282 | 0.014 | 3.060 | 0.547 |
| *Halomonas neptunia* | DSM15720 | 28 | 514 | 8 | 0.123 | 0.029 | 2.233 | 0.800 |
| *Halomonas sulfidaeris* | DSM15722 | 28 | 514 | 8 | 0.191 | 0.062 | 3.526 | 0.210 |
| *Halomonas axialensis* | DSM15723 | 28 | 514 | 8 | 0.345 | 0.056 | 3.528 | 0.075 |
| *Halomonas hydrothermalis* | DSM15725 | 28 | 514 | 8 | 0.537 | 0.151 | 3.486 | 0.260 |
| *Halomonas ventosae* | DSM15911 | 28 | 514 | 5 | 0.172 | 0.040 | 3.590 | 0.149 |
| *Halomonas glaciei* | DSM15938 | 22 | 514 | 2 | 0.184 | 0.043 | 2.213 | 0.183 |
| *Halomonas gudaonensis* | DSM23417 | 28 | 514 | 2 | 0.180 | 0.052 | 3.065 | 1.154 |
| *Halomonas anticariensis* | DSM16096 | 30 | 514 | 4.5 | 0.187 | 0.035 | 3.228 | 0.623 |
| *Halomonas organivorans* | DSM16226 | 37 | 514 | 8 | 0.158 | 0.059 | 3.167 | 0.246 |
| *Halomonas alkaliphila* | DSM16354 | 37 | 514 | 8 | 0.266 | 0.041 | 3.202 | 0.700 |
| *Halomonas taeanensis* | DSM16463 | 35 | 514 | 10 | 0.165 | 0.017 | 2.400 | 0.895 |
| *Halomonas kenyensis* | DSM17331 | 36 | 514 | 8 | 0.122 | 0.029 | 3.748 | 0.250 |
| *Halomonas mongoliensis* | DSM17332 | 36 | 514 | 8 | 0.203 | 0.072 | 1.867 | 0.233 |
| *Halomonas kribbensis* | DSM17892 | 33 | 514 | 5 | 0.104 | 0.041 | 1.847 | 0.211 |
| *Halomonas gomseomensis* | DSM18042 | 28 | 830 | 10 | 0.059 | 0.030 | 0.702 | 0.274 |
| *Halomonas janggokensis* | DSM18043 | 28 | 830 | 10 | 0.000 | 0.000 | 0.320 | 0.130 |
| *Halomonas denitrificans* | DSM18045 | 28 | 514 | 10 | 0.124 | 0.009 | 3.553 | 0.151 |
| *Halomonas caseinilytica* | DSM18067 | 37 | 372 | 20.2 | 0.391 | 0.183 | 1.603 | 0.768 |
| *Halomonas caseinilytica* | DSM23509 | 28 | 514 | 5 | 0.199 | 0.064 | 3.728 | 0.149 |
| *Halomonas olivaria* | DSM19074 | 37 | 514 | 8 | 0.171 | 0.079 | 3.247 | 0.230 |
| *Halomonas sabkhae* | DSM19122 | 40 | 514 | 10 | 0.160 | 0.082 | 2.148 | 0.747 |
| *Halomonas lutea* | DSM19221 | 37 | 514 | 10 | 0.178 | 0.038 | 3.835 | 0.245 |
| *Halomonas lutea* | DSM23508 | 37 | 514 | 5 | 0.209 | 0.055 | 2.091 | 0.626 |
| *Halomonas andesensis* | DSM19434 | 28 | 514 | 2 | 0.271 | 0.038 | 3.702 | 0.175 |
| *Halomonas korlensis* | DSM19633 | 28 | 514 | 10 | 0.158 | 0.033 | 3.477 | 0.320 |
| *Halomonas sediminis* | DSM19821 | 37 | 514 | 10 | 0.112 | 0.029 | 0.957 | 0.142 |
| *Halomonas ilicicola* | DSM19980 | 28 | 514 | 10 | 0.139 | 0.053 | 2.753 | 0.997 |
| *Halomonas vilamensis* | DSM21020 | 28 | 514 | 10 | 0.168 | 0.058 | 0.884 | 0.280 |
| *Halomonas zhanjiangensis* | DSM21076 | 28 | 514 | 5 | 0.152 | 0.034 | 3.321 | 0.481 |
| *Halomonas hamiltonii* | DSM21196 | 28 | 514 | 2 | 0.295 | 0.012 | 3.672 | 0.108 |
| *Halomonas johnsoniae* | DSM21197 | 28 | 514 | 2 | 0.278 | 0.058 | 2.329 | 0.479 |
| *Halomonas stevensii* | DSM21198 | 28 | 514 | 2 | 0.184 | 0.032 | 3.594 | 0.316 |
| *Halomonas smyrnensis* | DSM21644 | 37 | 514 | 10 | 0.192 | 0.033 | 3.659 | 0.158 |
| *Halomonas qijiaojingensis* | DSM22403 | 37 | 514 | 7 | 0.120 | 0.026 | 3.759 | 0.249 |
| *Halomonas urmiana* | DSM22871 | 28 | 514 | 2 | 0.512 | 0.124 | 1.285 | 0.224 |
| *Halomonas titanicae* | DSM22872 | 37 | 514 | 8 | 0.263 | 0.053 | 3.429 | 0.256 |
| *Halomonas tabrizica* | DSM23018 | 28 | 514 | 2 | 0.241 | 0.103 | 0.856 | 0.257 |
| *Halomonas shengliensis* | DSM23416 | 28 | 514 | 2 | 0.386 | 0.238 | 1.792 | 0.594 |
| *Halomonas nitroreducens* | DSM23437 | 37 | 514 | 5 | 0.269 | 0.042 | 2.959 | 0.804 |
| *Halomonas cerina* | DSM23438 | 28 | 514 | 8 | 0.189 | 0.024 | 2.704 | 0.916 |
| *Halomonas koreensis* | DSM23530 | 37 | 514 | 2 | 0.382 | 0.229 | 1.663 | 0.168 |
| *Halomonas saccharevitans* | DSM23548 | 28 | 514 | 2 | 0.242 | 0.060 | 1.088 | 0.174 |
| *Halomonas arcis* | DSM23549 | 28 | 514 | 2 | 0.113 | 0.037 | 3.762 | 0.157 |
| *Halomonas subterranea* | DSM23550 | 28 | 514 | 2 | 0.160 | 0.024 | 1.751 | 0.154 |
| *Halomonas almeriensis* | DSM23775 | 28 | 514 | 5 | 0.158 | 0.038 | 1.414 | 0.119 |
| *Halomonas nanhaiensis* | DSM25561 | 28 | 514 | 5 | 0.156 | 0.018 | 2.112 | 0.208 |
| *Halomonas lionensis* | DSM25632 | 28 | 514 | 2 | 0.224 | 0.037 | 1.031 | 0.131 |
| *Halomonas zhaodongensis* | DSM25869 | 37 | 514 | 7 | 0.174 | 0.049 | 3.777 | 0.212 |
| *Halomonas songnenensis* | DSM25870 | 37 | 514 | 5 | 0.212 | 0.039 | 3.643 | 0.256 |
| *Halomonas qiaohouensis* | DSM26770 | 28 | 514 | 2 | 0.264 | 0.031 | 3.394 | 0.624 |
| *Halomonas heilongjiangensis* | DSM26881 | 28 | 514 | 5 | 0.209 | 0.012 | 3.757 | 0.112 |
| *Halomonas lactosivorans* | DSM103220 | 28 | 514 | 2 | 0.204 | 0.045 | 3.658 | 0.264 |
| *Halomonas alkalicola* | DSM103354 | 28 | 514 | 2 | 0.228 | 0.032 | 3.694 | 0.231 |
| *Halomonas nigrificans* | DSM105749 | 30 | 514 | 2 | 0.327 | 0.164 | 3.500 | 0.000 |
| *Halomonas sedimenti* | DSM112091 | 30 | 514 | 2 | 0.283 | 0.030 | 3.750 | 0.217 |
| *Halomonas maris* | DSM112092 | 37 | 514 | 2 | 0.311 | 0.057 | 1.716 | 0.186 |
| *Halomonas eurialkalitoleransis* | ATCC  TSD-110 | 30 | TSD-110  Medium | 10 | 0.140 | 0.017 | 0.881 | 0.211 |
| *Halomonas halmophila* | DSM5349 | 25 | 514 | 10 | 0.030 | 0.006 | 2.074 | 0.235 |

**Table S6.** Mean specific growth rate (μ) and maximum OD_600_ of 23 *Halomonas* strains in their respectively recommended culture media at their reference growth temperature (n = 6, with standard deviation)

| ***Halomonas* species*** | **Strain ID** | **Reference growth temperature (ºC)** | **Recommended medium** | **Recommended [NaCl] (%)** | **μ (h^-1^)** | | **Maximum OD_600_** | |
| --- | --- | --- | --- | --- | --- | --- | --- | --- |
|  |  |  |  |  | **Average** | **Standard Deviation** | **Average** | **Standard Deviation** |
| *Halomonas sp.* | DSM7328 | 28 | 1428 | 10 | 0.246 | 0.056 | 2.198 | 0.177 |
| *Halomonas elongata* | DSM2581 | 28 | 514 | 2 | 0.457 | 0.252 | 3.424 | 0.171 |
| *Halomonas utahensis* | DSM3051 | 28 | 514 | 8 | 0.247 | 0.059 | 2.225 | 0.230 |
| *Halomonas aquamarina* | DSM30161 | 28 | 514 | 2 | 0.328 | 0.065 | 1.158 | 0.452 |
| *Halomonas cupida* | DSM4740 | 28 | 514 | 2 | 0.221 | 0.075 | 1.786 | 0.256 |
| *Halomonas venusta* | DSM4743 | 28 | 514 | 2 | 0.176 | 0.029 | 3.486 | 0.123 |
| *Halomonas pacifica* | DSM4742 | 28 | 514 | 2 | 0.277 | 0.075 | 3.485 | 0.219 |
| *Halomonas eurihalina* | DSM5720 | 28 | 1689 | 6.2 | 0.174 | 0.006 | 2.093 | 0.170 |
| *Halomonas salina* | DSM5928 | 28 | 514 | 8 | 0.278 | 0.027 | 2.282 | 0.264 |
| *Halomonas pantelleriensis* | DSM9661 | 28 | 2049 | 10.2 | 0.178 | 0.031 | 2.045 | 0.201 |
| *Halomonas halocynthiae* | DSM14573 | 28 | 514 | 2 | 0.303 | 0.038 | 2.295 | 0.426 |
| *Halomonas alimentaria* | DSM15356 | 28 | 514 | 2 | 0.131 | 0.028 | 3.494 | 0.305 |
| *Halomonas glaciei* | DSM15938 | 28 | 514 | 2 | 0.160 | 0.019 | 3.660 | 0.180 |
| *Halomonas anticariensis* | DSM16096 | 28 | 514 | 4.5 | 0.213 | 0.036 | 3.479 | 0.214 |
| *Halomonas taeanensis* | DSM16463 | 37 | 514 | 10 | 0.202 | 0.055 | 1.770 | 0.731 |
| *Halomonas kenyensis* | DSM17331 | 37 | 514 | 8 | 0.213 | 0.046 | 3.426 | 0.136 |
| *Halomonas mongoliensis* | DSM17332 | 37 | 514 | 8 | 0.149 | 0.039 | 1.061 | 0.223 |
| *Halomonas kribbensis* | DSM17892 | 28 | 514 | 5 | 0.230 | 0.090 | 1.723 | 0.246 |
| *Halomonas sabkhae* | DSM19122 | 37 | 514 | 10 | 0.127 | 0.025 | 2.998 | 0.698 |
| *Halomonas nigrificans* | DSM105749 | 28 | 514 | 2 | 0.285 | 0.060 | 3.566 | 0.269 |
| *Halomonas sedimenti* | DSM112091 | 28 | 514 | 2 | 0.245 | 0.041 | 3.109 | 0.688 |
| *Halomonas eurialkalitoleranis* | ATCC TSD-110 | 28 | TSD-110 medium | 10 | 0.128 | 0.029 | 1.317 | 0.271 |
| *Halomonas halmophila* | DSM5349 | 28 | 514 | 10 | 0.039 | 0.003 | 1.978 | 0.227 |

* Data presented here only belong to those species with a recommended growth Temperature reported to be different than 28^o^C or 37^o^C.

**Table S7.** Mean specific growth rate (μ) and maximum OD_600_ of 82 *Halomonas* strains grown in LB medium at their reference growth temperature (n = 6, with standard deviation)

| ***Halomonas* species** | **Strain ID** | **Reference growth temperature (ºC)** | **Recommended [NaCl] (%)** | **μ (h^-1^)** | | | **Maximum OD_600_** | | |
| --- | --- | --- | --- | --- | --- | --- | --- | --- | --- |
|  |  |  |  | **Average** | **Standard Deviation** | **Difference from recommended medium*** | **Average** | **Standard Deviation** | **Difference from recommended medium*** |
| *Halomonas sp.* | DSM7328 | 28 | 10 | 0.167 | 0.077 | Similar | 3.341 | 0.159 | Increase |
| *Halomonas elongata* | DSM2581 | 28 | 2 | 0.369 | 0.101 | Similar | 1.653 | 0.122 | Decrease |
| *Halomonas campaniensis* | DSM15293 | 37 | 10 | 0.397 | 0.065 | Similar | 3.317 | 0.558 | Similar |
| *Halomonas halophila* | DSM4770 | 28 | 10 | 0.285 | 0.231 | Similar | 1.620 | 0.331 | Decrease |
| *Halomonas boliviensis* | DSM15516 | 28 | 8 | 0.104 | 0.026 | Decrease | 2.540 | 0.807 | Similar |
| *Halomonas halodenitrificans* | DSM735 | 28 | 8 | 0.238 | 0.026 | Decrease | 3.222 | 0.314 | Similar |
| *Halomonas utahensis* | DSM3051 | 28 | 8 | 0.203 | 0.074 | Similar | 3.267 | 0.516 | Increase |
| *Halomonas subglaciescola* | DSM4683 | 28 | 8 | 0.053 | 0.028 | Decrease | 1.057 | 0.222 | Similar |
| *Halomonas aquamarina* | DSM30161 | 28 | 2 | 0.000 | 0.000 | Decrease | 0.200 | 0.097 | Decrease |
| *Halomonas cupida* | DSM4740 | 28 | 2 | 0.204 | 0.090 | Similar | 0.996 | 0.138 | Decrease |
| *Halomonas venusta* | DSM4743 | 28 | 2 | 0.231 | 0.035 | Similar | 2.306 | 0.756 | Similar |
| *Halomonas pacifica* | DSM4742 | 28 | 2 | 0.229 | 0.041 | Similar | 2.715 | 0.628 | Similar |
| *Halomonas meridiana* | DSM5425 | 28 | 2 | 0.254 | 0.044 | Similar | 2.715 | 0.628 | Decrease |
| *Halomonas eurihalina* | DSM5720 | 28 | 6.2 | 0.181 | 0.040 | Decrease | 3.125 | 0.875 | Similar |
| *Halomonas salina* | DSM5928 | 28 | 8 | 0.682 | 0.111 | Increase | 1.872 | 0.276 | Similar |
| *Halomonas desiderata* | DSM9502 | 28 | 2 | 0.124 | 0.023 | Decrease | 3.284 | 0.584 | Similar |
| *Halomonas pantelleriensis* | DSM9661 | 28 | 10.2 | 0.073 | 0.024 | Decrease | 3.546 | 0.212 | Increase |
| *Halomonas maura* | DSM13445 | 28 | 5 | 0.000 | 0.000 | Decrease | 0.250 | 0.220 | Decrease |
| *Halomonas halocynthiae* | DSM14573 | 28 | 2 | 0.301 | 0.065 | Similar | 1.495 | 0.206 | Decrease |
| *Halomonas muralis* | DSM14789 | 28 | 2 | 0.000 | 0.000 | Decrease | 0.147 | 0.115 | Decrease |
| *Halomonas alimentaria* | DSM15356 | 28 | 2 | 0.000 | 0.000 | Decrease | 0.384 | 0.211 | Decrease |
| *Halomonas magadiensis* | DSM15367 | 37 | 8 | 0.388 | 0.040 | Increase | 3.468 | 0.352 | Similar |
| *Halomonas campisalis* | DSM15413 | 28 | 2 | 0.121 | 0.019 | Decrease | 1.837 | 0.186 | Decrease |
| *Halomonas alkaliantarctica* | DSM15686 | 28 | 10 | 0.204 | 0.062 | Similar | 3.838 | 0.153 | Increase |
| *Halomonas neptunia* | DSM15720 | 28 | 8 | 0.115 | 0.047 | Similar | 0.975 | 0.096 | Decrease |
| *Halomonas sulfidaeris* | DSM15722 | 28 | 8 | 0.119 | 0.032 | Similar | 3.226 | 0.221 | Similar |
| *Halomonas axialensis* | DSM15723 | 28 | 8 | 0.253 | 0.100 | Similar | 2.430 | 0.652 | Decrease |
| *Halomonas hydrothermalis* | DSM15725 | 28 | 8 | 0.288 | 0.103 | Decrease | 3.143 | 0.606 | Similar |
| *Halomonas ventosae* | DSM15911 | 28 | 5 | 0.139 | 0.067 | Similar | 1.917 | 0.192 | Decrease |
| *Halomonas glaciei* | DSM15938 | 28 | 2 | 0.113 | 0.032 | Decrease | 2.788 | 0.727 | Similar |
| *Halomonas gudaonensis* | DSM23417 | 28 | 2 | 0.000 | 0.000 | Decrease | 0.115 | 0.072 | Decrease |
| *Halomonas anticariensis* | DSM16096 | 28 | 4.5 | 0.124 | 0.025 | Decrease | 3.029 | 0.943 | Similar |
| *Halomonas organivorans* | DSM16226 | 37 | 8 | 0.234 | 0.057 | Similar | 3.671 | 0.187 | Increase |
| *Halomonas alkaliphila* | DSM16354 | 37 | 8 | 0.287 | 0.032 | Similar | 3.405 | 0.112 | Similar |
| *Halomonas taeanensis* | DSM16463 | 37 | 10 | 0.156 | 0.087 | Similar | 2.523 | 0.924 | Similar |
| *Halomonas kenyensis* | DSM17331 | 37 | 8 | 0.158 | 0.039 | Similar | 3.498 | 0.116 | Similar |
| *Halomonas mongoliensis* | DSM17332 | 37 | 8 | 0.406 | 0.101 | Increase | 3.483 | 0.331 | Increase |
| *Halomonas kribbensis* | DSM17892 | 37 | 5 | 0.000 | 0.000 | Decrease | 0.181 | 0.048 | Decrease |
| *Halomonas gomseomensis* | DSM18042 | 28 | 10 | 0.170 | 0.042 | Increase | 1.529 | 0.347 | Increase |
| *Halomonas janggokensis* | DSM18043 | 28 | 10 | 0.288 | 0.106 | Increase | 2.455 | 0.848 | Increase |
| *Halomonas denitrificans* | DSM18045 | 28 | 10 | 0.233 | 0.099 | Similar | 1.844 | 0.182 | Decrease |
| *Halomonas caseinilytica* | DSM18067 | 37 | 20.2 | 0.125 | 0.034 | Decrease | 1.600 | 0.094 | Similar |
| *Halomonas caseinilytica* | DSM23509 | 28 | 5 | 0.142 | 0.029 | Similar | 1.805 | 0.220 | Decrease |
| *Halomonas olivaria* | DSM19074 | 37 | 8 | 0.141 | 0.030 | Similar | 1.707 | 0.417 | Decrease |
| *Halomonas sabkhae* | DSM19122 | 37 | 10 | 0.137 | 0.100 | Similar | 1.737 | 0.219 | Similar |
| *Halomonas lutea* | DSM19221 | 37 | 10 | 0.108 | 0.035 | Decrease | 1.525 | 0.492 | Decrease |
| *Halomonas lutea* | DSM23508 | 37 | 5 | 0.000 | 0.000 | Decrease | 0.231 | 0.161 | Decrease |
| *Halomonas andesensis* | DSM19434 | 28 | 2 | 0.232 | 0.046 | Similar | 2.279 | 0.539 | Decrease |
| *Halomonas korlensis* | DSM19633 | 28 | 10 | 0.072 | 0.015 | Decrease | 1.964 | 0.289 | Decrease |
| *Halomonas sediminis* | DSM19821 | 28 | 10 | 0.050 | 0.020 | Decrease | 0.971 | 0.200 | Similar |
| *Halomonas ilicicola* | DSM19980 | 28 | 10 | 0.000 | 0.000 | Decrease | 0.115 | 0.129 | Decrease |
| *Halomonas vilamensis* | DSM21020 | 28 | 10 | 0.062 | 0.025 | Decrease | 0.367 | 0.207 | Decrease |
| *Halomonas zhanjiangensis* | DSM21076 | 28 | 5 | 0.251 | 0.054 | Increase | 2.954 | 0.699 | Similar |
| *Halomonas hamiltonii* | DSM21196 | 28 | 2 | 0.070 | 0.017 | Decrease | 2.455 | 0.824 | Decrease |
| *Halomonas johnsoniae* | DSM21197 | 28 | 2 | 0.195 | 0.048 | Similar | 2.832 | 0.757 | Similar |
| *Halomonas stevensii* | DSM21198 | 28 | 2 | 0.620 | 0.277 | Increase | 1.919 | 0.207 | Decrease |
| *Halomonas smyrnensis* | DSM21644 | 37 | 10 | 0.166 | 0.048 | Similar | 1.493 | 0.595 | Decrease |
| *Halomonas qijiaojingensis* | DSM22403 | 37 | 7 | 0.168 | 0.063 | Similar | 3.095 | 0.910 | Similar |
| *Halomonas urmiana* | DSM22871 | 28 | 2 | 0.000 | 0.000 | Decrease | 0.148 | 0.067 | Decrease |
| *Halomonas titanicae* | DSM22872 | 37 | 8 | 0.189 | 0.048 | Similar | 3.092 | 0.588 | Similar |
| *Halomonas tabrizica* | DSM23018 | 28 | 2 | 0.000 | 0.000 | Decrease | 0.173 | 0.148 | Decrease |
| *Halomonas shengliensis* | DSM23416 | 28 | 2 | 0.000 | 0.000 | Decrease | 0.348 | 0.145 | Decrease |
| *Halomonas nitroreducens* | DSM23437 | 37 | 5 | 0.000 | 0.000 | Decrease | 0.424 | 0.165 | Decrease |
| *Halomonas cerina* | DSM23438 | 28 | 8 | 0.115 | 0.036 | Decrease | 1.250 | 0.287 | Decrease |
| *Halomonas koreensis* | DSM23530 | 37 | 2 | 0.000 | 0.000 | Decrease | 0.225 | 0.333 | Decrease |
| *Halomonas saccharevitans* | DSM23548 | 28 | 2 | 0.000 | 0.000 | Decrease | 0.929 | 0.553 | Decrease |
| *Halomonas arcis* | DSM23549 | 28 | 2 | 0.000 | 0.000 | Decrease | 0.322 | 0.143 | Decrease |
| *Halomonas subterranea* | DSM23550 | 28 | 2 | 0.108 | 0.013 | Decrease | 1.405 | 0.202 | Decrease |
| *Halomonas almeriensis* | DSM23775 | 28 | 5 | 0.000 | 0.000 | Decrease | 0.594 | 0.266 | Decrease |
| *Halomonas nanhaiensis* | DSM25561 | 28 | 5 | 0.272 | 0.085 | Increase | 1.977 | 0.369 | Similar |
| *Halomonas lionensis* | DSM25632 | 28 | 2 | 0.380 | 0.198 | Similar | 2.512 | 0.757 | Increase |
| *Halomonas zhaodongensis* | DSM25869 | 37 | 7 | 0.240 | 0.060 | Similar | 3.299 | 0.218 | Decrease |
| *Halomonas songnenensis* | DSM25870 | 37 | 5 | 0.210 | 0.028 | Similar | 3.554 | 0.326 | Similar |
| *Halomonas qiaohouensis* | DSM26770 | 28 | 2 | 0.270 | 0.052 | Similar | 1.862 | 0.180 | Decrease |
| *Halomonas heilongjiangensis* | DSM26881 | 28 | 5 | 0.326 | 0.074 | Increase | 3.519 | 0.242 | Similar |
| *Halomonas lactosivorans* | DSM103220 | 28 | 2 | 0.252 | 0.031 | Similar | 3.549 | 0.104 | Similar |
| *Halomonas alkalicola* | DSM103354 | 28 | 2 | 0.141 | 0.024 | Decrease | 3.375 | 0.557 | Similar |
| *Halomonas nigrificans* | DSM105749 | 28 | 2 | 0.182 | 0.015 | Similar | 1.299 | 0.176 | Decrease |
| *Halomonas sedimenti* | DSM112091 | 28 | 2 | 0.157 | 0.010 | Decrease | 3.478 | 0.168 | Similar |
| *Halomonas maris* | DSM112092 | 37 | 2 | 0.000 | 0.000 | Decrease | 0.249 | 0.154 | Decrease |
| *Halomonas eurialkalitoleransis* | ATCC TSD-110 | 28 | 10 | 0.191 | 0.050 | Similar | 3.763 | 0.345 | Increase |
| *Halomonas halmophila* | DSM5349 | 28 | 10 | 0.126 | 0.015 | Increase | 3.166 | 0.729 | Increase |

^* denotes a significant difference in the respective column (p < 0.01)^

**Table S8.** Mean specific growth rate (μ) and maximum OD_600_ of 82 *Halomonas* strains in LB medium supplemented with 8% (LB80) or 2% (LB20) NaCl at their reference growth temperature (n = 6, with standard deviation)

| ***Halomonas* species** | **Strain ID** | **μ (h^-1^)** | | | | **Maximum OD_600_** | | | |
| --- | --- | --- | --- | --- | --- | --- | --- | --- | --- |
|  |  | **LB80** | | **LB20** | | **LB80** | | **LB20** | |
|  |  | **Average** | **SD*** | **Average** | **SD*** | **Average** | **SD*** | **Average** | **SD*** |
| *Halomonas sp.* | DSM7328 | 0.267 | 0.070 | 0.100 | 0.023 | 3.534 | 0.169 | 2.861 | 0.569 |
| *Halomonas elongata* | DSM2581 | 0.305 | 0.076 | 0.369 | 0.101 | 2.686 | 0.781 | 1.653 | 0.122 |
| *Halomonas campaniensis* | DSM15293 | 0.362 | 0.150 | 0.291 | 0.126 | 3.329 | 0.633 | 1.708 | 0.243 |
| *Halomonas halophila* | DSM4770 | 0.179 | 0.044 | 0.259 | 0.045 | 1.577 | 0.333 | 1.912 | 0.117 |
| *Halomonas boliviensis* | DSM15516 | 0.104 | 0.026 | 0.178 | 0.085 | 2.540 | 0.807 | 1.807 | 0.359 |
| *Halomonas halodenitrificans* | DSM735 | 0.238 | 0.026 | 0.000 | 0.000 | 3.222 | 0.314 | 0.545 | 0.218 |
| *Halomonas utahensis* | DSM3051 | 0.203 | 0.074 | 0.259 | 0.104 | 3.267 | 0.516 | 1.640 | 0.360 |
| *Halomonas subglaciescola* | DSM4683 | 0.053 | 0.028 | 0.000 | 0.000 | 1.057 | 0.222 | 0.219 | 0.200 |
| *Halomonas aquamarina* | DSM30161 | 0.189 | 0.118 | 0.000 | 0.000 | 3.523 | 0.312 | 0.200 | 0.097 |
| *Halomonas cupida* | DSM4740 | 0.503 | 0.195 | 0.204 | 0.090 | 3.517 | 0.250 | 0.996 | 0.138 |
| *Halomonas venusta* | DSM4743 | 0.226 | 0.032 | 0.231 | 0.035 | 3.439 | 0.182 | 2.306 | 0.756 |
| *Halomonas pacifica* | DSM4742 | 0.067 | 0.025 | 0.229 | 0.041 | 2.399 | 0.915 | 2.715 | 0.628 |
| *Halomonas meridiana* | DSM5425 | 0.133 | 0.094 | 0.254 | 0.044 | 3.424 | 0.678 | 2.715 | 0.628 |
| *Halomonas eurihalina* | DSM5720 | 0.293 | 0.074 | 0.191 | 0.042 | 3.258 | 0.691 | 2.552 | 0.777 |
| *Halomonas salina* | DSM5928 | 0.682 | 0.111 | 0.087 | 0.024 | 1.872 | 0.276 | 1.768 | 0.122 |
| *Halomonas desiderata* | DSM9502 | 0.087 | 0.030 | 0.124 | 0.023 | 2.097 | 0.945 | 3.284 | 0.584 |
| *Halomonas pantelleriensis* | DSM9661 | 0.088 | 0.011 | 0.197 | 0.035 | 3.327 | 0.211 | 1.819 | 0.259 |
| *Halomonas maura* | DSM13445 | 0.000 | 0.000 | 0.000 | 0.000 | 0.191 | 0.116 | 0.360 | 0.235 |
| *Halomonas halocynthiae* | DSM14573 | 0.224 | 0.090 | 0.301 | 0.065 | 3.408 | 0.168 | 1.495 | 0.206 |
| *Halomonas muralis* | DSM14789 | 0.000 | 0.000 | 0.000 | 0.000 | 0.190 | 0.122 | 0.147 | 0.115 |
| *Halomonas alimentaria* | DSM15356 | 0.097 | 0.009 | 0.000 | 0.000 | 3.769 | 0.138 | 0.384 | 0.211 |
| *Halomonas magadiensis* | DSM15367 | 0.388 | 0.040 | 0.170 | 0.024 | 3.468 | 0.352 | 2.467 | 0.620 |
| *Halomonas campisalis* | DSM15413 | 0.071 | 0.014 | 0.121 | 0.019 | 2.911 | 0.985 | 1.837 | 0.186 |
| *Halomonas alkaliantarctica* | DSM15686 | 0.236 | 0.070 | 0.296 | 0.098 | 3.562 | 0.161 | 1.469 | 0.225 |
| *Halomonas neptunia* | DSM15720 | 0.115 | 0.047 | 0.080 | 0.021 | 0.975 | 0.096 | 1.667 | 0.533 |
| *Halomonas sulfidaeris* | DSM15722 | 0.119 | 0.032 | 0.000 | 0.000 | 3.226 | 0.221 | 0.229 | 0.142 |
| *Halomonas axialensis* | DSM15723 | 0.253 | 0.100 | 0.250 | 0.074 | 2.430 | 0.652 | 1.868 | 0.270 |
| *Halomonas hydrothermalis* | DSM15725 | 0.288 | 0.103 | 0.556 | 0.195 | 3.143 | 0.606 | 2.447 | 0.631 |
| *Halomonas ventosae* | DSM15911 | 0.205 | 0.119 | 0.000 | 0.000 | 3.149 | 0.479 | 0.567 | 0.122 |
| *Halomonas glaciei* | DSM15938 | 0.075 | 0.043 | 0.113 | 0.032 | 3.764 | 0.322 | 2.788 | 0.727 |
| *Halomonas gudaonensis* | DSM23417 | 0.088 | 0.016 | 0.000 | 0.000 | 3.248 | 0.878 | 0.115 | 0.072 |
| *Halomonas anticariensis* | DSM16096 | 0.152 | 0.052 | 0.000 | 0.000 | 2.694 | 0.796 | 0.370 | 0.299 |
| *Halomonas organivorans* | DSM16226 | 0.234 | 0.057 | 0.000 | 0.000 | 3.671 | 0.187 | 0.166 | 0.104 |
| *Halomonas alkaliphila* | DSM16354 | 0.287 | 0.032 | 0.202 | 0.062 | 3.405 | 0.112 | 3.778 | 0.214 |
| *Halomonas taeanensis* | DSM16463 | 0.226 | 0.144 | 0.000 | 0.000 | 2.069 | 0.821 | 0.407 | 0.205 |
| *Halomonas kenyensis* | DSM17331 | 0.158 | 0.039 | 0.145 | 0.022 | 3.498 | 0.116 | 3.205 | 0.676 |
| *Halomonas mongoliensis* | DSM17332 | 0.406 | 0.101 | 0.317 | 0.036 | 3.483 | 0.331 | 2.850 | 0.650 |
| *Halomonas kribbensis* | DSM17892 | 0.000 | 0.000 | 0.000 | 0.000 | 1.193 | 0.539 | 1.006 | 0.352 |
| *Halomonas gomseomensis* | DSM18042 | 0.249 | 0.040 | 0.000 | 0.000 | 3.755 | 0.251 | 0.140 | 0.100 |
| *Halomonas janggokensis* | DSM18043 | 0.220 | 0.035 | 0.000 | 0.000 | 3.677 | 0.139 | 0.229 | 0.189 |
| *Halomonas denitrificans* | DSM18045 | 0.151 | 0.090 | 0.156 | 0.027 | 2.283 | 0.962 | 3.407 | 0.317 |
| *Halomonas caseinilytica* | DSM18067 | 0.100 | 0.022 | 0.063 | 0.015 | 2.603 | 0.834 | 1.055 | 0.100 |
| *Halomonas caseinilytica* | DSM23509 | 0.133 | 0.015 | 0.094 | 0.020 | 1.278 | 0.203 | 1.429 | 0.206 |
| *Halomonas olivaria* | DSM19074 | 0.141 | 0.030 | 0.242 | 0.101 | 1.707 | 0.417 | 1.803 | 0.131 |
| *Halomonas sabkhae* | DSM19122 | 0.221 | 0.116 | 0.000 | 0.000 | 2.835 | 0.901 | 0.419 | 0.248 |
| *Halomonas lutea* | DSM19221 | 0.106 | 0.067 | 0.000 | 0.000 | 1.129 | 0.387 | 0.261 | 0.116 |
| *Halomonas lutea* | DSM23508 | 0.097 | 0.017 | 0.000 | 0.000 | 1.787 | 0.212 | 0.236 | 0.149 |
| *Halomonas andesensis* | DSM19434 | 0.158 | 0.030 | 0.232 | 0.046 | 2.676 | 0.547 | 2.279 | 0.539 |
| *Halomonas korlensis* | DSM19633 | 0.092 | 0.021 | 0.000 | 0.000 | 3.194 | 0.718 | 0.231 | 0.142 |
| *Halomonas sediminis* | DSM19821 | 0.085 | 0.017 | 0.000 | 0.000 | 1.751 | 0.255 | 0.148 | 0.125 |
| *Halomonas ilicicola* | DSM19980 | 0.055 | 0.014 | 0.000 | 0.000 | 3.554 | 0.164 | 0.729 | 0.295 |
| *Halomonas vilamensis* | DSM21020 | 0.130 | 0.030 | 0.000 | 0.000 | 1.883 | 0.236 | 0.254 | 0.128 |
| *Halomonas zhanjiangensis* | DSM21076 | 0.288 | 0.052 | 0.275 | 0.093 | 3.325 | 0.316 | 3.570 | 0.235 |
| *Halomonas hamiltonii* | DSM21196 | 0.181 | 0.098 | 0.070 | 0.017 | 2.156 | 0.600 | 2.455 | 0.824 |
| *Halomonas johnsoniae* | DSM21197 | 0.187 | 0.052 | 0.195 | 0.048 | 2.704 | 0.525 | 2.832 | 0.757 |
| *Halomonas stevensii* | DSM21198 | 0.123 | 0.014 | 0.620 | 0.277 | 3.423 | 0.225 | 1.919 | 0.207 |
| *Halomonas smyrnensis* | DSM21644 | 0.098 | 0.012 | 0.000 | 0.000 | 1.450 | 0.330 | 0.172 | 0.051 |
| *Halomonas qijiaojingensis* | DSM22403 | 0.138 | 0.101 | 0.176 | 0.034 | 3.616 | 0.254 | 1.777 | 0.323 |
| *Halomonas urmiana* | DSM22871 | 0.250 | 0.094 | 0.000 | 0.000 | 3.589 | 0.277 | 0.148 | 0.067 |
| *Halomonas titanicae* | DSM22872 | 0.189 | 0.048 | 0.074 | 0.046 | 3.092 | 0.588 | 1.555 | 0.146 |
| *Halomonas tabrizica* | DSM23018 | 0.000 | 0.000 | 0.000 | 0.000 | 0.210 | 0.086 | 0.173 | 0.148 |
| *Halomonas shengliensis* | DSM23416 | 0.160 | 0.041 | 0.000 | 0.000 | 2.594 | 0.659 | 0.348 | 0.145 |
| *Halomonas nitroreducens* | DSM23437 | 0.125 | 0.044 | 0.000 | 0.000 | 2.704 | 0.924 | 0.266 | 0.173 |
| *Halomonas cerina* | DSM23438 | 0.115 | 0.036 | 0.000 | 0.000 | 1.250 | 0.287 | 0.185 | 0.146 |
| *Halomonas koreensis* | DSM23530 | 0.092 | 0.034 | 0.000 | 0.000 | 2.100 | 0.164 | 0.225 | 0.333 |
| *Halomonas saccharevitans* | DSM23548 | 0.185 | 0.035 | 0.000 | 0.000 | 2.724 | 0.718 | 0.929 | 0.553 |
| *Halomonas arcis* | DSM23549 | 0.154 | 0.025 | 0.000 | 0.000 | 2.246 | 1.074 | 0.322 | 0.143 |
| *Halomonas subterranea* | DSM23550 | 0.131 | 0.027 | 0.108 | 0.013 | 2.749 | 0.701 | 1.405 | 0.202 |
| *Halomonas almeriensis* | DSM23775 | 0.147 | 0.042 | 0.000 | 0.000 | 1.544 | 0.241 | 0.701 | 0.344 |
| *Halomonas nanhaiensis* | DSM25561 | 0.169 | 0.060 | 0.130 | 0.037 | 2.803 | 0.692 | 2.401 | 0.869 |
| *Halomonas lionensis* | DSM25632 | 0.049 | 0.025 | 0.380 | 0.198 | 0.741 | 0.123 | 2.512 | 0.757 |
| *Halomonas zhaodongensis* | DSM25869 | 0.311 | 0.114 | 0.292 | 0.065 | 3.588 | 0.192 | 3.512 | 0.253 |
| *Halomonas songnenensis* | DSM25870 | 0.316 | 0.032 | 0.519 | 0.056 | 3.620 | 0.135 | 2.836 | 0.787 |
| *Halomonas qiaohouensis* | DSM26770 | 0.255 | 0.025 | 0.270 | 0.052 | 2.483 | 0.642 | 1.862 | 0.180 |
| *Halomonas heilongjiangensis* | DSM26881 | 0.361 | 0.091 | 0.354 | 0.078 | 3.221 | 0.071 | 3.014 | 0.476 |
| *Halomonas lactosivorans* | DSM103220 | 0.273 | 0.094 | 0.252 | 0.031 | 3.500 | 0.168 | 3.549 | 0.104 |
| *Halomonas alkalicola* | DSM103354 | 0.205 | 0.024 | 0.141 | 0.024 | 3.217 | 0.396 | 3.375 | 0.557 |
| *Halomonas nigrificans* | DSM105749 | 0.165 | 0.061 | 0.182 | 0.015 | 1.694 | 1.138 | 1.299 | 0.176 |
| *Halomonas sedimenti* | DSM112091 | 0.125 | 0.015 | 0.157 | 0.010 | 3.428 | 0.240 | 3.478 | 0.168 |
| *Halomonas maris* | DSM112092 | 0.307 | 0.029 | 0.000 | 0.000 | 3.238 | 0.614 | 0.249 | 0.154 |
| *Halomonas eurialkalitoleransis* | ATCC  TSD-110 | 0.326 | 0.094 | 0.458 | 0.103 | 3.399 | 0.345 | 1.901 | 0.302 |
| *Halomonas halmophila* | DSM5349 | 0.076 | 0.043 | 0.000 | 0.000 | 1.252 | 0.404 | 0.161 | 0.067 |

* denotes standard deviation.

**Table S9.** Summary of reference growth conditions for 82 *Halomonas* strains

| ***Halomonas* species** | **Strain ID** | **Culture-collection recommended conditions** | | | **Reference condition in LB**** | |
| --- | --- | --- | --- | --- | --- | --- |
|  |  | **RM*** | **Temperature (°C)** | **[NaCl] (%)** | **Temperature (°C)** | **[NaCl] (%)** |
| *Halomonas* sp. | DSM7328 | 1428 | 30 | 10.1 | 28 | 8 |
| *Halomonas elongata* | DSM2581 | 514 | 30 | 2 | 28 | 8 |
| *Halomonas campaniensis* | DSM15293 | 1034 | 37 | 10 | 37 | 8 |
| *Halomonas halophila* | DSM4770 | 514 | 28 | 10 | 28 | 2 |
| *Halomonas boliviensis* | DSM15516 | 514 | 28 | 8 | 28 | 2,8 |
| *Halomonas halodenitrificans* | DSM735 | 514 | 28 | 8 | 28 | 8 ^a^ |
| *Halomonas utahensis* | DSM3051 | 514 | 30 | 8 | 28 | 8 |
| *Halomonas subglaciescola* | DSM4683 | 514 | 28 | 8 | 28 | 8 ^a^ |
| *Halomonas aquamarina* | DSM30161 | 514 | 25 | 2 | 28 | 8 ^a^ |
| *Halomonas cupida* | DSM4740 | 514 | 25 | 2 | 28 | 8 |
| *Halomonas venusta* | DSM4743 | 514 | 25 | 2 | 28 | 8 |
| *Halomonas pacifica* | DSM4742 | 514 | 26 | 2 | 28 | 2 |
| *Halomonas meridiana* | DSM5425 | 514 | 28 | 2 | 28 | 2 |
| *Halomonas eurihalina* | DSM5720 | 1689 | 30 | 6.2 | 28 | 8 |
| *Halomonas salina* | DSM5928 | 514 | 30 | 8 | 28 | 8 |
| *Halomonas desiderata* | DSM9502 | 514 | 28 | 2 | 28 | 2 |
| *Halomonas pantelleriensis* | DSM9661 | 2049 | 30 | 10.2 | 28 | 2,8 ^b^ |
| *Halomonas maura* | DSM13445 | 514 | 28 | 5 | N/A | |
| *Halomonas halocynthiae* | DSM14573 | 514 | 25 | 2 | 28 | 2,8 ^b^ |
| *Halomonas muralis* | DSM14789 | 514 | 28 | 2 | N/A | |
| *Halomonas alimentaria* | DSM15356 | 514 | 30 | 2 | 28 | 8 ^a^ |
| *Halomonas magadiensis* | DSM15367 | 514 | 37 | 8 | 37 | 8 |
| *Halomonas campisalis* | DSM15413 | 514 | 28 | 2 | 28 | 2,8 ^b^ |
| *Halomonas alkaliantarctica* | DSM15686 | 514 | 28 | 10 | 28 | 8 |
| *Halomonas neptunia* | DSM15720 | 514 | 28 | 8 | 28 | 2 |
| *Halomonas sulfidaeris* | DSM15722 | 514 | 28 | 8 | 28 | 8 ^a^ |
| *Halomonas axialensis* | DSM15723 | 514 | 28 | 8 | 28 | 2,8 |
| *Halomonas hydrothermalis* | DSM15725 | 514 | 28 | 8 | 28 | 2 |
| *Halomonas ventosae* | DSM15911 | 514 | 28 | 5 | 28 | 8 ^a^ |
| *Halomonas glaciei* | DSM15938 | 514 | 22 | 2 | 28 | 8 |
| *Halomonas gudaonensis* | DSM23417 | 514 | 28 | 2 | 28 | 8 ^a^ |
| *Halomonas anticariensis* | DSM16096 | 514 | 30 | 4.5 | 28 | 8 ^a^ |
| *Halomonas organivorans* | DSM16226 | 514 | 37 | 8 | 37 | 8 ^a^ |
| *Halomonas alkaliphila* | DSM16354 | 514 | 37 | 8 | 37 | 2,8 ^c^ |
| *Halomonas taeanensis* | DSM16463 | 514 | 35 | 10 | 37 | 8 ^a^ |
| *Halomonas kenyensis* | DSM17331 | 514 | 36 | 8 | 37 | 2,8 |
| *Halomonas mongoliensis* | DSM17332 | 514 | 36 | 8 | 37 | 2,8 |
| *Halomonas kribbensis* | DSM17892 | 514 | 33 | 5 | N/A | |
| *Halomonas gomseomensis* | DSM18042 | 830 | 28 | 10 | 28 | 8 ^a^ |
| *Halomonas janggokensis* | DSM18043 | 830 | 28 | 10 | 28 | 8 ^a^ |
| *Halomonas denitrificans* | DSM18045 | 514 | 28 | 10 | 28 | 2 |
| *Halomonas caseinilytica* | DSM18067 | 372 | 37 | 20.2 | 37 | 8 |
| *Halomonas caseinilytica* | DSM23509 | 514 | 28 | 5 | 28 | 8 |
| *Halomonas olivaria* | DSM19074 | 514 | 37 | 8 | 37 | 2 |
| *Halomonas sabkhae* | DSM19122 | 514 | 40 | 10 | 37 | 8 ^a^ |
| *Halomonas lutea* | DSM19221 | 514 | 37 | 10 | 37 | 8 ^a^ |
| *Halomonas lutea* | DSM23508 | 514 | 37 | 5 | 37 | 8 ^a^ |
| *Halomonas andesensis* | DSM19434 | 514 | 28 | 2 | 28 | 2 |
| *Halomonas korlensis* | DSM19633 | 514 | 28 | 10 | 28 | 8 ^a^ |
| *Halomonas sediminis* | DSM19821 | 514 | 37 | 10 | 28 | 8 ^a^ |
| *Halomonas ilicicola* | DSM19980 | 514 | 28 | 10 | 28 | 8 ^a^ |
| *Halomonas vilamensis* | DSM21020 | 514 | 28 | 10 | 28 | 8 ^a^ |
| *Halomonas zhanjiangensis* | DSM21076 | 514 | 28 | 5 | 28 | 2,8 |
| *Halomonas hamiltonii* | DSM21196 | 514 | 28 | 2 | 28 | 8 |
| *Halomonas johnsoniae* | DSM21197 | 514 | 28 | 2 | 28 | 2,8 |
| *Halomonas stevensii* | DSM21198 | 514 | 28 | 2 | 28 | 2,8 ^b^ |
| *Halomonas smyrnensis* | DSM21644 | 514 | 37 | 10 | 37 | 8 ^a^ |
| *Halomonas qijiaojingensis* | DSM22403 | 514 | 37 | 7 | 37 | 8 |
| *Halomonas urmiana* | DSM22871 | 514 | 28 | 2 | 28 | 8 ^a^ |
| *Halomonas titanicae* | DSM22872 | 514 | 37 | 8 | 37 | 8 |
| *Halomonas tabrizica* | DSM23018 | 514 | 28 | 2 | N/A | |
| *Halomonas shengliensis* | DSM23416 | 514 | 28 | 2 | 28 | 8 ^a^ |
| *Halomonas nitroreducens* | DSM23437 | 514 | 37 | 5 | 37 | 8 ^a^ |
| *Halomonas cerina* | DSM23438 | 514 | 28 | 8 | 28 | 8 ^a^ |
| *Halomonas koreensis* | DSM23530 | 514 | 37 | 2 | 37 | 8 ^a^ |
| *Halomonas saccharevitans* | DSM23548 | 514 | 28 | 2 | 28 | 8 ^a^ |
| *Halomonas arcis* | DSM23549 | 514 | 28 | 2 | 28 | 8 ^a^ |
| *Halomonas subterranea* | DSM23550 | 514 | 28 | 2 | 28 | 8 |
| *Halomonas almeriensis* | DSM23775 | 514 | 28 | 5 | 28 | 8 ^a^ |
| *Halomonas nanhaiensis* | DSM25561 | 514 | 28 | 5 | 28 | 2,8 |
| *Halomonas lionensis* | DSM25632 | 514 | 28 | 2 | 28 | 2 |
| *Halomonas zhaodongensis* | DSM25869 | 514 | 37 | 7 | 37 | 2,8 |
| *Halomonas songnenensis* | DSM25870 | 514 | 37 | 5 | 37 | 2,8 ^b^ |
| *Halomonas qiaohouensis* | DSM26770 | 514 | 28 | 2 | 28 | 8 |
| *Halomonas heilongjiangensis* | DSM26881 | 514 | 28 | 5 | 28 | 2,8 |
| *Halomonas lactosivorans* | DSM103220 | 514 | 28 | 2 | 28 | 2,8 |
| *Halomonas alkalicola* | DSM103354 | 514 | 28 | 2 | 28 | 8 |
| *Halomonas nigrificans* | DSM105749 | 514 | 30 | 2 | 28 | 2,8 |
| *Halomonas sedimenti* | DSM112091 | 514 | 30 | 2 | 28 | 2 |
| *Halomonas maris* | DSM112092 | 514 | 37 | 2 | 37 | 8 ^a^ |
| *Halomonas eurialkalitoleransis* | ATCC TSD-110 | TSD-110 Medium | 30 | 10 | 28 | 2,8 ^b^ |
| *Halomonas halmophila* | DSM5349 | 514 | 25 | 10 | 28 | 8 ^a^ |

^*^ Medium 514 refers to both BD Difco and Merck Marine broth 2216 as reported in DSMZ website.

^**^ N/A: Not available

^a^ denotes exclusive growth in 2% or 8% salt.

^b^ denotes ability to grow in either 2% or 8% salt concentration, with higher specific growth rate when cultured in LB20 than LB80, and higher OD_max_ when cultured in LB80 than LB20.

^c^ denotes ability to grow in either 2% or 8% salt concentration, with higher specific growth rate when cultured in LB80 than LB20, and higher OD_max_ when cultured in LB20 than LB80.

**Supplementary Figures**

**Figure S1.** The relationship between phylogenetic tree based on 16S rRNA sequences of *Halomonas* strains and their culture-collection recommended (A) temperature, and (B) NaCl concentration. Recommended temperature and NaCl concentration were represented using a colour gradient ranging from 40 °C (Dark green) to 22 °C (White), and from 20.2% (Dark green) to 2% (White), respectively. The different culturing recommendations for two *Halomonas* *caseinilytica* strains DSM18067 and DSM23509 is highlighted in the lower red square. A similar difference observed for *Halomonas* *lutea* (DSM19221 identified by a red square) could not be shown on the phylogenetic tree as the 16S rRNA sequence is not available for the non-type strain DSM 23508 for the species, with a recommended growth temperature of 37 °C and a salt concentration of 5%.

**Figure S2.** Growth profiles of *H. alimentaria* DSM15356 in recommended culture medium (Marine broth 2216 (Millipore)) grown at 30 and 28 °C which were the collection-recommended temperature (Blue) and the reference temperature (Orange) of the strain, respectively.

**Figure S3.** Tanglegram constructed of 60 *Halomonas* strains between the 16S rRNA-based phylogenetic tree (Left) and growth characteristic (specific growth rates and maximum OD_600_) clusters of recommended culture conditions (Right).

**Figure S4.** Tanglegram constructed of 60 *Halomonas* strains between the 16S rRNA-based phylogenetic tree (Left) and growth characteristic (specific growth rates and maximum OD_600_) clusters of reference culture conditions (Right).

**Supplementary Analysis S1**

**1. Phylogenetic Tree Construction**

**1.1 Computational Environment, Data Acquisition and Cleaning**

A dataset of 79 16S rRNA sequences was compiled from public databases (*accession numbers provided in Supplementary Table S3*). Core sequence standardisation, sequence alignment, alignment trimming and maximum likelihood phylogenetic reconstructions were executed on Amazon Web Services (AWS) Elastic Compute Cloud (EC2) instance running Linux. Downstream phenotypic data standardisation, multivariate clustering, phylogenetic signal testing and high-resolution figure rendering were performed within R studio integrated development environment (IDE) under a Windows 10 x64, using R (v.4.5.1) via R studio (v2025.9.1.401) (*see Supplementary Table S4 for relevant packages and versions).* Initial data cleaning was performed to ensure a non-redundant dataset. Duplicate entries, identified by identical genetic accessions or redundant file naming conventions (e.g., "(2)" suffixes), were removed, retaining exactly one representative sequence per accession. To ensure computational compatibility and figure clarity, FASTA headers were standardised to a unique ACCESSION|Taxonomic_Label format (e.g., *AJ306889|Halomonas_halmophila_DSM5349*). This pre-processing step ensured that all downstream branch labels were unique and human readable.

**1.2 Multiple Sequence Alignment (MSA) and Trimming**

A total of 79 curated sequences (representing 78 species) were consolidated into a single multi-FASTA file for construction of the phylogenetic tree. Two strains (*Halomonas sp.* DSM7328 and *H. eurialkalitoleransis* TSD-110) were excluded from phylogenetic comparison analyses due to the absence of 16S rRNA sequences in the reference dataset*. H. lutea* DSM23508 shares a phylogenetic representative with the other *H. lutea* stain in this collection DSM19221 rather than being independently excluded.

Sequence alignment was performed using *MAFFT* (v.7.0.0), utilising its Fast Fourier Transform (FFT) heuristics and iterative refinement to align homologous nucleotide positions across the 16S rRNA gene. To mitigate the influence of phylogenetic noise originating from hypervariable loops and alignment gaps, the MSA was processed with *trimAl* (v.1.5.0). Unreliable or gap-heavy columns were removed to produce a refined alignment suitable for maximum-likelihood inference.

**1.3 Phylogenetic Inference**

Phylogenetic trees were reconstructed using the Maximum Likelihood (ML) criteria via IQ-TREE (v.3.0.1). The software’s ModelFinder module was employed to select the optimal DNA substitution model, accounting for base frequencies, invariable sites, and rate heterogeneity across sites. Branch support was assessed using two concurrent metrics: the SH-like approximate likelihood ratio test (SH-aLRT) and the Ultrafast Bootstrap (UFBoot) with 1,000 replicates. Following standard protocols, support was considered strong when SH-aLRT was greater than or equal to 80% and UFBoot was greater than or equal to 95%.

**1.4 Rooting and Visualisation**

The resulting ML phylogeny was midpoint-rooted using the *ape* and *phangorn* packages within the R statistical environment. This method was selected to provide an objective root in the absence of a predefined outgroup. Rooting stability was subsequently confirmed by comparison against explicit outgroup rooting using *Zymobacter palmae,* which recovered an identical topology (Robinson Foulds Distance= 0, 80 or 80 non-root internal splits identical. The final tree was exported for high-resolution visualisation, with branch lengths representing the estimated number of nucleotide substitutions per site.

**2. Hierarchical clustering**

All input variables were scaled to unit variance prior to clustering using the scale function in R to ensure equal contribution of variables measured on different scales. Clustering was performed using Ward's minimum variance method (ward.D2) with Euclidean distance calculated, implemented via the *hclust* function. The optimal number of clusters (k) was determined independently for each analysis primarily using the average silhouette width, which measures the cohesion and separation of clusters with higher values indicating better-defined groupings (Rousseeuw, 1987); the value of k corresponding to the maximum average silhouette width. Where silhouette increased monotonically towards the upper bound of the range tested (k=10) with no interior maximum, this was treated as an indication of overfitting into small poorly separated clusters rather than genuine structure; in these cases, k was instead selected using the elbow method, which plots total within-cluster sum of squares (WSS) against k and identifies the point of inflection. Both methods were implemented using the *cluster* package. Where both methods indicated the same k that value was used directly. Cluster membership and mean variable values per cluster were exported for biological interpretation. Dendrograms were visualised using the *dendextend* package (Galili, 2015) and exported as TIFF figures at 300 dpi.

**3. Phylogenetic Signal Testing**

To assess whether phenotypic growth traits are phylogenetically conserved, three complementary analyses were performed for each growth condition comparison across 79 matched strains representing 78 unique species present in both the laboratory dataset and the 16S rRNA phylogenetic tree. (i) Tanglegram analysis was performed using the *dendextend* package, placing the 16S rRNA phylogeny alongside phenotypic clustering dendrograms connected by lines linking the same strain on both trees. The degree of discordance between trees was quantified using the entanglement score, ranging from 0 (perfect agreement) to 1 (complete mismatch). Entanglement was calculated using the entanglement function. Prior to tanglegram construction, the phylogenetic tree was converted to an ultrametric tree using the chronos function from the *ape* package (Paradis and Schliep, 2019), which adjusts branch lengths while preserving topology, a step required for dendrogram conversion. (ii) Mantel tests were performed using the mantel function from the *vegan* package (Oksanen et al., 2022) with 9,999 permutations and Pearson correlation. The Mantel test formally tests the correlation between phylogenetic distance matrices (derived from cophenetic distances of the ultrametric tree) and phenotypic distance matrices (Euclidean distances of scaled trait values). A significant result (p < 0.05) indicates that strains more closely related evolutionarily also tend to be more phenotypically similar. The Mantel test was chosen over regression-based approaches because it directly compares distance matrices without assuming a specific functional relationship between phylogenetic and phenotypic distance. (iii) Blomberg's K and Pagel's λ were calculated for each individual trait using the *phylosig* function from the *phytools* package (Revell, 2012). Both statistics test whether trait variation across strains is more phylogenetically structured than expected under a Brownian motion model of trait evolution. Blomberg's K = 1 indicates evolution consistent with Brownian motion; K > 1 indicates stronger phylogenetic conservatism than expected; K < 1 indicates weaker conservatism. Pagel's λ ranges from 0 (no phylogenetic signal) to 1 (trait evolves in proportion to shared evolutionary history). Statistical significance was assessed using 999 randomisation permutations for Blomberg's K and likelihood ratio tests for Pagel's λ. Both statistics were calculated because they are sensitive to different aspects of phylogenetic signal: Blomberg's K is more sensitive to signal across the entire tree while Pagel's λ is more sensitive to signal in recent divergences. Blomberg's K was selected over alternative statistics such as Moran's I because it directly accounts for the phylogenetic covariance structure and is robust to small sample sizes (Blomberg et al., 2003). Single-condition tanglegram and Mantel analyses were additionally performed for each individual growth variable to detect condition-specific phylogenetic signal that may be masked in combined analyses.

**4. Cluster validity for growth characteristic clustering**

Cluster validity was assessed using MANOVA on the joint distribution of specific growth rate and maximum OD_600_ for the 60 strains assigned a unique reference conditions. As expected, given that clustering was performed directly on these variables clusters differed significantly under both collection recommended and proposed reference conditions (Wilks' Λ = 0.092 and 0.141, respectively; both p < 2.2×10⁻¹⁶). This confirms that the clustering itself was statistically well separated but does not constitute independent evidence against a genuine biological pattern.

To confirm that these clusters reflect a genuine biological patter rather than simply restating how they were built, cluster membership under the proposed reference conditions was tested against each strains country of isolation, grouped as China vs. all as other countries as it was the only grouping with adequate sample size, no significant association was found (Fishers exact test, p =0.27) indicating the clustering is not simply an artefect of the variables used to define it.

**Supplementary Text S1:**

**Standard Operating Procedure for cultivation of an uncharacterised *Halomonas* isolate using the reference decision rule**

**Materials**

- LB medium base
- NaCl, for supplementation to 8% (w/v) (LB80) and 2% (w/v) (LB20)
- Glycerol stock of the isolate (or equivalent starting culture)
- 96-well microplate (e.g., SARSTEDT Cat. No. 82.1581)
- Gas-permeable plate seal (e.g., Aeraseal™, EXCEL Cat. No. Z721573)
- Plate reader capable of continuous absorbance monitoring with orbital shaking (e.g., CLARIOstar Plus, BMG Labtech)
- Shaking incubator (for preculture step, if separate from the plate reader)

**Procedure**

1. Select the default condition. Prepare LB medium supplemented to 8% NaCl (LB80). Determine the incubation temperature by selecting whichever of 28°C or 37°C is closer to the isolate's known or presumed isolation temperature.
2. Prepare the preculture. Revive the isolate from glycerol stock by inoculating into LB80 medium. Incubate overnight with shaking at the selected temperature. If no visible growth (turbidity) is observed after overnight incubation, do not discard the culture; continue incubation for up to 14 days, checking periodically, before concluding that the isolate cannot be revived under this condition. Once a healthy, turbid preculture is obtained, proceed to Step 3; the extended incubation window is not required again once cells are established from precultures.
3. Standardise the inoculum. Measure the OD₆₀₀ of the preculture. Dilute into fresh LB80 medium to achieve an initial OD₆₀₀ of 0.05.
4. Dispense into the culture plate. Aliquot 200 μL of the standardised culture per well into a 96-well microplate. Prepare 6 biological replicate wells per strain-condition combination. In parallel wells on the same plate, include uninoculated LB80 medium blanks for background subtraction.
5. Seal the plate. Cover the plate with a gas-permeable seal (e.g., Aeraseal™). This seal type is used specifically to maximise aeration capacity, as oxygen transfer can vary with the salt composition of the medium.
6. Incubate and monitor. Place the sealed plate in a plate reader set to the selected temperature, with rotational agitation of 200 rpm (double orbital shaking). Record OD₆₀₀ readings at regular intervals — every 5 minutes for runs expected to last up to approximately 82 hours, or every 10 minutes for runs expected to extend to approximately 136 hours. Continue monitoring for a minimum of 46 hours, and until stationary phase is reached or the planned run duration elapses. pH and aeration are not actively controlled during this step.
7. Correct the raw data. For each well, normalise the first OD₆₀₀ reading to 0.05, treating this as an additive offset, and apply the same offset to all subsequent readings from that well. Subtract the corresponding medium blank (measured in parallel on the same plate) from each corrected reading.
8. Determine the exponential phase and calculate growth rate. Natural-log-transform the blank-corrected OD₆₀₀ values and plot against incubation time. Identify the linear region of this plot corresponding to the exponential growth phase, ensuring the selected window spans at least two hours. Calculate the specific growth rate (μ) as the slope of this linear region, and record the maximum OD₆₀₀ (OD_max) observed over the full run.
9. Assess growth under LB80. Using the calculated growth curve, determine whether the isolate exhibits discernible exponential growth under LB80. If growth is established, this condition (LB80, with the selected temperature) is taken as the reference cultivation condition for the isolate, and the procedure ends here.
10. Fall back to LB20 if growth is not established. If the isolate does not exhibit discernible growth under LB80, repeat Steps 2–9 using LB medium supplemented to 2% NaCl (LB20) in place of LB80, at the same temperature.
11. Record the final outcome. Report the condition (LB80 or LB20, with the temperature used) under which growth was established. If the isolate fails to grow under both LB80 and LB20 within the tested timeframe, record it as falling outside the two-condition framework and requiring strain-specific optimisation.
